# Criteria for unmet need in paediatric populations and their families: a literature-based case study in haematological malignancies in upper-middle and high-income countries

**DOI:** 10.3389/fped.2026.1765938

**Published:** 2026-02-13

**Authors:** Charlotte Van Isterdael, Zilke Claessens, Isabelle Huys

**Affiliations:** 1 Department of Pharmaceutical and Pharmacological Sciences, KU Leuven, Leuven, Belgium; 2 Research Foundation Flanders, Brussel, Belgium

**Keywords:** family needs, hematological malignances, pediatric population, scoping review, unmet needs

## Abstract

**Introduction:**

Unmet health-related needs (UHNs) in paediatric populations are often under-researched due to methodological challenges. Yet, understanding these UHNs is essential to improve health outcomes and quality of life for children. Importantly, their families also bear a substantial burden throughout the disease trajectory. This study (i) identifies UHN criteria specific to children and families, (ii) compare them with criteria included in the KCE NEED framework, and (iii) maps the methodological tools used to assess these needs.

**Methods:**

A scoping review was conducted following JBI methodology and reported using PRISMA-ScR. Selection of sources of evidence was conducted double-blind. UHNs were thematically analysed across health, healthcare and social domains, focusing on children with haematologic malignancies as a representative population. Studies were solely included if conducted in upper-middle and high-income countries.

**Results:**

The review (*n* = 34) identified 17 UHN criteria experienced by children or their families. The criteria varied by child's age: in older groups UHNs related to treatment adherence, age-dependent information and involvement, and future perspectives, while across all ages, challenges with educational continuity, mental health, social interactions, forgone care and autonomy were mentioned. UHN for families relate to family and financial stability, mental health, social interactions, information needs, care organization, and privacy. Specific to siblings, parental attention was identified. A total of 14 tools were identified, comprising 8 tools to assess patients’ UHNs, and 6 on their families’ UHNs.

**Discussion:**

The multifaceted nature of UHNs in children with haematologic malignancies and their families calls for a holistic, age-appropriate approach to care. These findings may be extrapolated to broader paediatric populations, highlighting the need for age-dependent communication, sustained educational support, and comprehensive family-centred care.

**Systematic Review Registration:**

https://osf.io/xfqyw/overview.

## 1 Introduction

The concept of unmet medical need (UMN) has received significant attention in the recent proposal for the revision of the pharmaceutical legislation in Europe, where it is presented as a key concept to guide pharmaceutical research and development (R&D) towards addressing gaps in underserved areas, aiming to create a more patient-centred health system (1). Beyond the European regulatory context, national health agencies are also increasingly considering the application of the UMN concept in their decision-making processes. So does the Belgian regulator, who uses the UMN concept as an eligibility criterion for the application of early access schemes such as the medical need program (2). However, translating UMN into national frameworks has raised questions about scope and relevance of this concept in different contexts (3, 4).

To address this, the Belgian Health Care Knowledge Centre (KCE) has developed the Needs Evaluations, Examination and Dissemination (NEED) framework to systematically assess unmet health-related needs (UHNs) (5). This framework provides a structured and scientifically robust methodology for identifying patient and societal needs through predefined criteria, indicators, using patient surveys, interviews with patients and experts, database analysis, and literature reviews (5). The ultimate goal of the NEED assessment is to ensure that healthcare stakeholders and policymakers integrate these findings into decision-making and priority-setting to enhance patient-centred care and healthcare innovation (5).

Although research has already been performed in identifying UHNs in adult patients, the needs of specific populations, such as the paediatric population, tend to be less frequently investigated. This is particularly striking given that, according to Eurostat in 2021, 4.4% of children reported a disability, defined as an activity limitations, due to health problems (6). The considerable differences in the pharmacokinetics and pharmacodynamics between children and adults highlight the risk of extrapolating adult data for paediatric use, risking underdosing or toxicity effects in those children (7–9).

Cancer is the primary cause of disease-related deaths among children and adolescents, with in 2022, approximately 14,000 cancer diagnoses in children in Europe, and around 2,000 deaths reported annually (10). The importance of research on children is shown in acute lymphoblast leukaemia, where the five-year survival rate spectacularly improved from 25% to 75% following multicentre trials including children, instead of extrapolating adult data for use in children (8).

The objectives of this study are (i) to identify and characterise paediatric-specific unmet health needs (UHN) criteria, (ii) to explore if these UHN criteria are already included in the existing KCE NEED framework and provide recommendations for tailoring the framework for paediatric-specific application, and (iii) to identify existing tools used to assess these needs in the paediatric population.

Haematologic malignancies were chosen for this study due to their high prevalence, accounting for more than one-third of paediatric malignancies, ensuring generalisable findings (10). The disease can occur across all paediatric age groups, allowing for insights into age-specific health needs. Its early onset and persistence throughout childhood further enable an assessment of UHNs across different developmental stages. Additionally, intensive and prolonged treatments, including chemotherapy, radiation and stem cell transplantation, lead to long-term physical, psychological, and social challenges. This broad impact makes haematologic malignancies an ideal model for studying UHNs in paediatric chronic illness.

## 2 Methods

### 2.1 Guidelines

A scoping review on UHNs for research in the paediatric population was conducted in accordance with the JBI methodology for scoping reviews (11). The PRISMA extension for scoping reviews was used for reporting (12).

### 2.2 Data sources and search strategy

Electronic searches were performed in two scientific databases, Pubmed (including MEDLINE; 1946–2024) and Embase (1974–2024). The search was performed on 5 March 2024. The four search concepts were methodologies, UHNs, paediatrics, and haematologic malignancies, each comprising index terms (MeSH–terms and Emtree-terms respectively) and other free text words to search in title, abstract, and keywords (Supplementary Information I). Before the start of the study, a protocol was registered in the Center for Open Science (13).

### 2.3 Eligibility and study selection

After article retrieval, duplicate articles were removed and a double-blinded pilot title and abstract (tiab) assessment against eligibility criteria (Table 1) on 10% of the articles was performed by two researchers (CV and ZC), with conflicts being resolved through discussion. The remaining 90% of the articles were assessed against the eligibility criteria, in a double-blinded manner on tiab by two researchers (CV, ZC) in the first phase with any uncertainties or doubts being discussed among the researchers to ensure consistency in the selection process. De-duplication and tiab screening were performed non-automated using Rayyan, a software for managing literature reviews. In the second phase, full-text articles were assessed against the eligibility criteria by two researchers (CV, ZC). Any disagreement that arose between the reviewers was resolved through discussion. Retrospective and prospective primary studies (e.g., cohort studies, cross-sectional studies, and case studies), and reviews examining UHNs of the paediatric population with haematologic malignancies, their family or informal caregivers were included.

**Table 1:** Inclusion- and exclusion criteria considered in the scoping literature review.

Criteria	Inclusion	Exclusion
Population	Studies focused on children^a^ (0–18yo) living with haematologic malignancies, their family, and/or informal caregivers or survivors of paediatric haematologic malignancies reflecting on their needs as a child.	Studies focused on adult patients, health-care providers or the broader society.
Outcomes	Studies conducting a health-related needs assessment of patients, survivors, their families and/or informal caregivers.	Studies that do not conduct a health-related needs assessment.
Study design	– Primary research: Retrospective or prospective studies on paediatric populations. – Secondary research: Systematic, scoping, or narrative reviews addressing methodological aspects.	– Studies without a methodological focus related to paediatric research, such as case reports, editorials, commentaries, and opinion pieces. – Clinical trials
Geographical region	Upper-middle- and high-income countries according to world population review (14)	Low- and lower-middle-income countries according to world population review (14)

a The paediatric population can be divided into younger children (0–11yo), adolescents (12–18yo), adolescents and young adults (AYA) (12–24yo).

### 2.4 Data extraction, analysis and synthesis

Data were collected using a predefined extraction framework, developed based on the NEED assessment framework (version dated 9/09/2024), relevant literature, and researchers' discussions (CV, ZC, IH) (Supplementary Information II) (5). Additionally, new themes that emerged during the extraction process were incorporated. For each included study, descriptive data (e.g., study title, authors, year, and geographic region) and content parameters (e.g., methods used, participant characteristics and UHNs) were extracted by one reviewer (CV) and cross-checked against the original articles by the second reviewer (ZC).

A descriptive analysis of the included studies was conducted independently by two reviewers (CV and ZC). First, methodological tools to identify and measure UHNs in paediatric patients were identified and listed. UHNs were thematically analysed and grouped into UHN criteria categories. These categories could overlap with the ones already included in the NEED framework but also extend beyond the existing criteria. Although all identified UHNs were extracted, only those specifically relevant or particularly pressing for the paediatric population and their families were discussed in detail in this review. We labelled criteria that were predominantly reported in AYA studies as “AYA-specific” to avoid generalisation to younger children. The identified criteria were compared with the KCE NEED framework (version of February 2025), an updated version of the framework as published in KCE Report 377C1 and existing generic criteria were evaluated for their relevance in the paediatric population.

## 3 Results

### 3.1 Overview of selected studies

**Figure 1:**
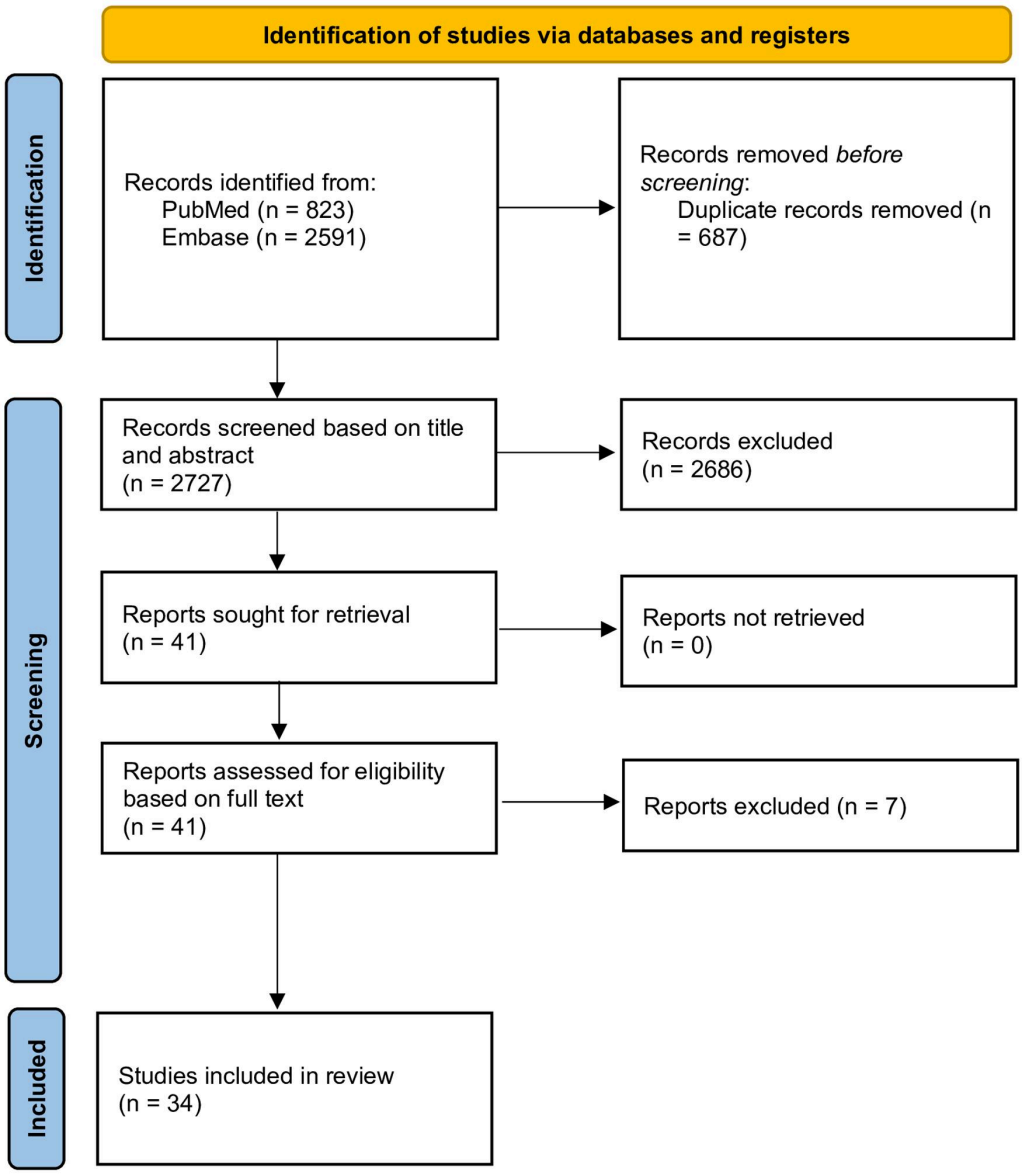
PRISMA flowchart of the identification of studies in the scoping literature review, covering identification, screening based on title and abstract as well as full text, and final inclusion of studies for analysis in the scoping review.

After screening and assessment for eligibility, 34 studies were included for analysis (Figure 1). A summary of articles included in the review is provided in Supplementary Information III.

**Figure 2:**
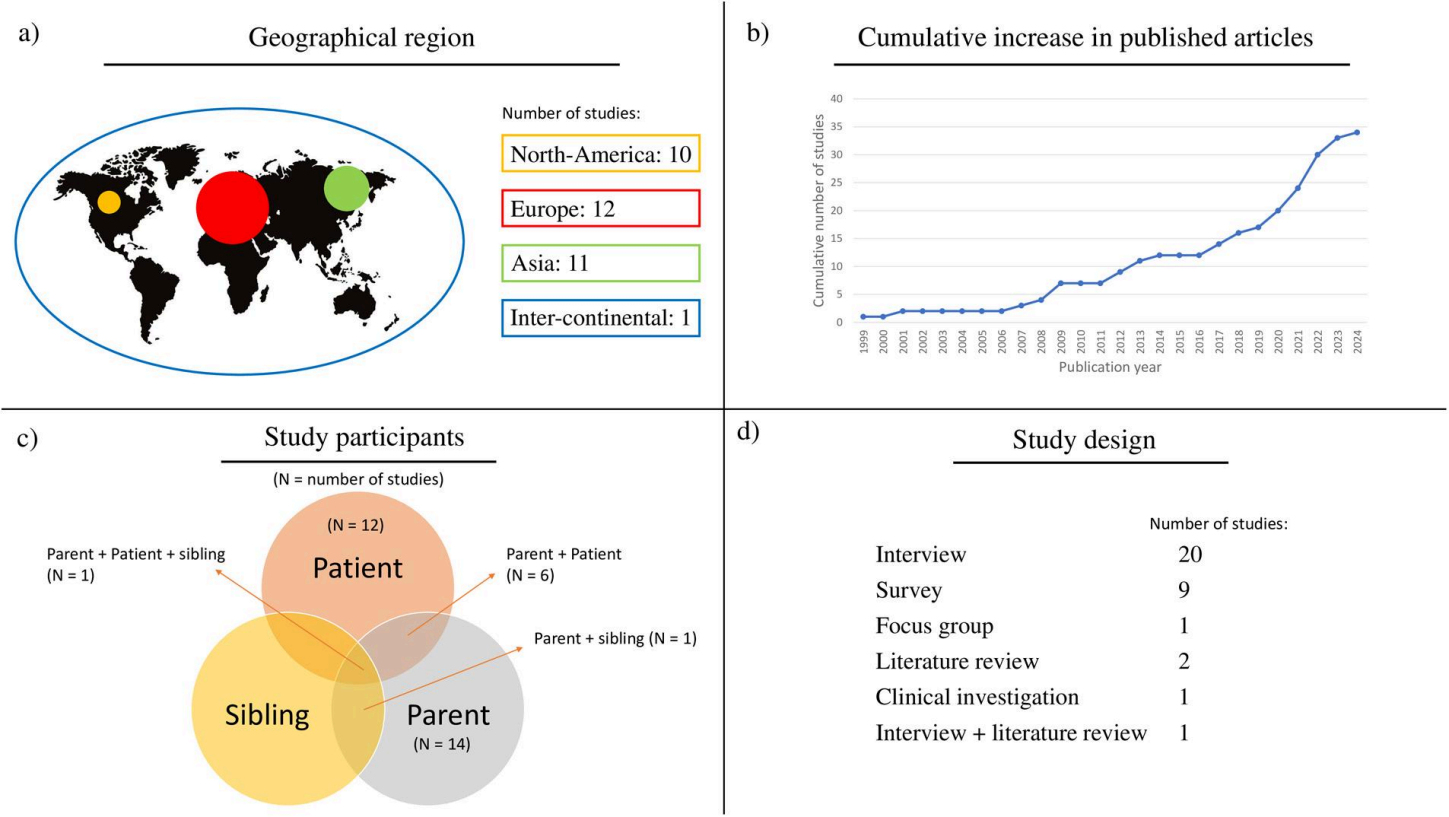
Descriptive data regarding the included studies. The geographical distribution of included articles is presented, showing a focus on Europe, but also other HMICs and HICs **(a)**. Moreover, it is shown that all relevant participant categories are covered by the included studies **(c)**. Finally, it provides an overview of published articles, clearly showing that in recent years there has been more attention for UHN research **(b)**, and the study design of the included studies being predominantly interview studies **(d)**.

Figure 2 presents the descriptive data of the included studies in terms of (a) geographical region, (b) year of publication, (c) study participants, and (d) study design. Most studies were performed in Europe (*N* = 12) and North America (*N* = 10), and 80% was conducted between 2012 and 2024 (*N* = 27). Most of the studies were interview studies (*N* = 20) and surveys (*N* = 9), with some studies using validated tools to identify UHNs in the paediatric population.

Table 2 provides an overview of all 14 tools that were used to systematically assess patients' UHNs. Specifically, 14 tools were identified, which can be classified into two overarching groups to investigate UHNs of either paediatric patients (*n* = 8) or their families (*n* = 6). Of the tools investigating patient UHNs, six were tools developed for children, with three tools addressing the children themselves and three tools using parents as a proxy to investigate UHNs in children. Two tools were adult tools used in children, one of which was modified for use in children.

**Table 2:** Tools to identify UHNs in paediatric haematological malignancies patients. Notably, most identified instruments focused on either patient self-report or parent-proxy outcomes, while few tools explicitly captured sibling needs or family-wide functioning, indicating a gap for comprehensive family-system assessment.

Tool	Whose needs assessed	Respondent group	Child-specific tool?
Tools directed to assess patients’ UHN			
Youth self-report (YSR) (15)	Paediatric patients	Paediatric patients	Yes—designed for paediatric use
The strength and difficulties questionnaire (SDQ) (15)	Paediatric patients	Paediatric patients	Yes—designed for paediatric use
AYA HOPE survey (16)	Paediatric patients^a^	Paediatric patients^a^	Yes—designed for paediatric use
Parental Account of Children's Symptoms (PACS) (15)	Paediatric patients	Parents	Yes—designed for paediatric use
Child behaviour checklist (CBCL) (15)	Paediatric patients	Parents	Yes—designed for paediatric use
HOPE Needs Assessment (17)	Paediatric patients	Parents	Yes—designed for paediatric use
Problem Need Palliative Care Questionnaire (PNPC) (18)	Paediatric patients	Paediatric patients	No—adult tool adapted for paediatric use
General Health Questionnaire (GHQ) (15)	Paediatric patients	Paediatric patients	No—generic tool
Tools directed to assess families’ UHN			
Perceived stress scale-10 (PSS-10) (19)	Parents	Parents	NA
Mini-COPE (19)	Parents	Parents	NA
ISEL-40 GP (19)	Parents	Parents	NA
Comprehensive Needs of Caregivers of Cancer Patients and Families taking care of Children Scale (20)	Parents	Parents	NA
Needs Assessment of Family Caregivers Cancer (NAFC-C) (21)	Parents	Parents	NA
Family adaptability and cohesion scale (FACES IV) (22)	Families (patient, siblings, parents)	Families (patient, siblings, parents)	NA

a Designed for adolescents and young adults, not younger children.

### 3.2 Criteria to evaluate health-related needs in paediatric patients

**Figure 3:**
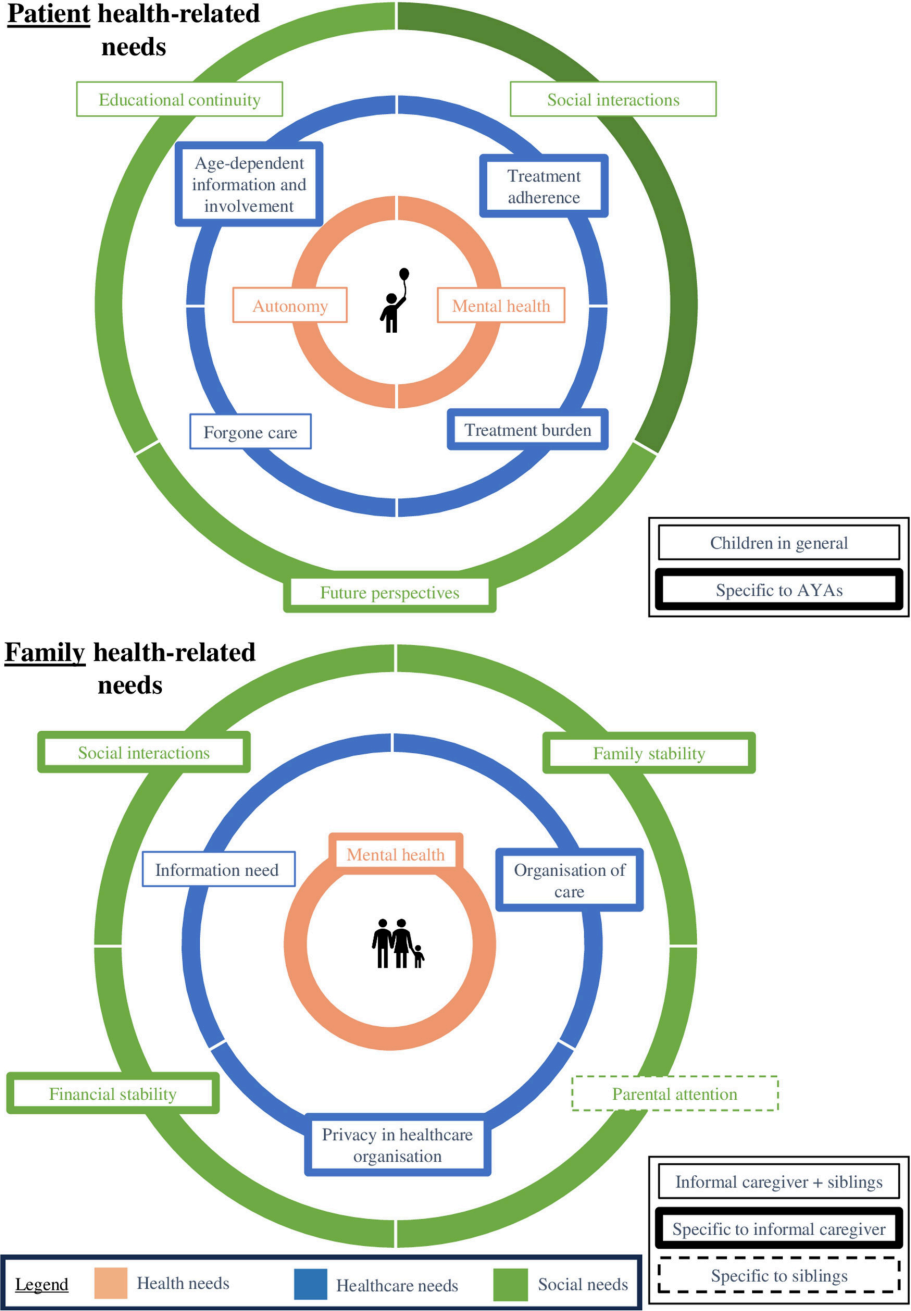
Overview of patient- and family- health-related need criteria in paediatric haematologic malignancies, organised across health, healthcare, and social domains to support comparison with the KCE NEED framework.

This section provides an overview of criteria capturing UHNs of children living with haematologic malignancies and their families over three domains: health, healthcare, and social, as presented in Figure 3 and detailed in the following paragraphs.

#### 3.2.1 Patient level

At the patient level, UHNs were identified across all age categories, although their presentation sometimes varied by age group, and certain needs were unique to specific age categories. To address these differences, age-specific criteria were developed to distinguish between the needs of AYAs (age-dependent information and involvement, treatment adherence, treatment burden and future perspectives) and children in general (educational continuity, social interactions, forgone care, autonomy and mental health).

a) Health needs: this domain includes two need criteria. The first criterion is *autonomy*, its disruption in younger children was reported in one study (18). Moreover, while pivotal for their life stage, gaining independency is often disrupted in adolescent patients with haematologic malignancies, resulting in concerns about becoming too weak and losing self-reliance (23–27). The second criterion is *mental health*, as children of all ages, but particularly AYAs, experience frustration and depression, specifically related to not being able to do things they used to do and over their appearance, as well as anxiety and emotional distress concerning overcoming cancer (28–33).
b) Healthcare needs: this domain includes four need criteria. By far the most frequently described criterion in this review was the need for *age-dependent information and involvement* of patients in examinations and decision-making. While parents often tend to limit involvement based on a child's age and maturity, children prefer open communication, as it fosters a better understanding of their situation and strengthens the relationship with their parents and healthcare professionals (HCPs) (34, 35). Particularly AYAs expressed a strong desire for clear, individualized, and trustworthy information about their diagnosis, treatment and prognosis, as well as collaboration in treatment decision-making (16, 23, 32, 33, 36–38). Another regularly reported unmet information need among AYAs concerns intimacy, fertility, and romantic relationships (32, 39–41). These conversations are often hindered by barriers such as discomfort and privacy concerns (16, 32, 39–43).
  The second criterion is *treatment adherence*, which is reported to be especially challenging for AYAs, as the treatment regimens considerably disrupt their daily activities and parental supervision is less consistent compared to that of younger children (23). The third criterion is *forgone care*, described in three articles. with other health-related needs, especially dental care, being neglected due to the already burdensome management of haematologic malignancies (44–46). The last criterion is the *burden of the treatment*. Children with haematologic malignancies face disturbing side effects that impact their daily activities and well-being and contribute to emotional distress (25, 30, 32, 37). Painful procedures evoke fear, with some children requiring sedation or anaesthesia for relief (37).
c) Social needs: this domain includes three need criteria. The first criterion is *educational continuity*. While some paediatric haematologic malignancies patients manage to study through remote assignments, many are unable to attend school in any form due to hospitalization, fatigue, or therapy side effects (17, 23, 27, 28, 31, 32, 36, 37, 43, 47–49). The second criterion is impact on *social interactions***,** as the illness disrupts friendships and social activities, leaving many feeling isolated, missing out on life experiences, and longing to “fit in” (25, 27, 28, 31, 32, 37, 38, 43, 48). The last criterion is specific to AYA, who often face a loss of *future perspective***,** leaving them concerned about career opportunities, financial stability, and marriage prospects (18, 23, 31).

#### 3.2.2 Family level

At the family level, a distinction was made between need criteria specific to informal caregivers and those specific to siblings of patients with haematological malignancies, or both.

a) Health needs: This domain includes one criterion: the impact on *mental health*. Multiple articles reported several symptoms experienced by informal caregivers, such as anxiety, stress, depression, the shock of diagnosis, and being overwhelmed by the feeling of responsibility for their child's recovery (15, 18–20, 30, 34, 35, 39, 47, 50–54). Causes of anxiety include uncertainty about the child's recovery and relapse, risk of infections, and specific fears related to daily caregiving tasks (18, 39, 51, 53).
b) Healthcare needs: this domain includes three need criteria. The first criterion is *information need***,** for both parents and siblings, which was reported in three articles. Parents regularly reported to lack information about the disease and how to care for their sick children (21, 22, 26, 35, 55, 56), but also siblings sometimes tend to require more information (22, 52). The second criterion, specifically for informal caregivers, is the *organisation of care*, including the need to cluster appointments, and continuity of care, which is reported to be a challenge as appointments are frequently handled by different HCPs (36, 57). The third criterion is also specific to informal caregivers and covers the need for *privacy* in hospitals, allowing parents to have private conversations with their children and with HCPs (36, 42).
c) Social needs: this domain includes four need criteria. The first criterion, experienced by informal caregivers, is the impact on *social interactions*. Some studies reported parents frequently giving up their social life and leisure activities to care for their sick children (29, 30, 47, 52). The second criterion is the impact on *family stability*, with parents reporting to often feel the need to mask their anxiety to the rest of their family, to appear strong for their family (34, 54). A third criterion is the impact on *financial stability* reported in seven articles, primarily caused by parents working less to take care of their sick child (18, 20, 29, 35, 38, 47, 53, 58). The fourth criterion is *reduced parental attention*. While this is experienced by the siblings, this is also acknowledged by parents who feel guilty for not spending equally much time with their healthy children (30, 35, 41, 47).

### 3.3 Comparison of paediatric-specific criteria identified in the literature with the generic KCE NEED framework

Table 3 provides a comparison between the findings of this review and the KCE NEED framework. Certain criteria of the KCE NEED framework, such as mental health support, educational continuity, and autonomy, were frequently reported and highly relevant in paediatric populations, while others, like impact on life expectancy, and treatment effectiveness, are only occasionally reported but still considered relevant. Additionally, some adult-relevant criteria, including work-related challenges, were not relevant for paediatric settings, highlighting the need for paediatric-specific adaptations of the framework.

**Table 3:** Comparison of the KCE NEED framework criteria with findings from the literature review. The criteria and definitions presented are based on the KCE NEED framework (version of February 2025), an updated version of the framework as published in KCE Report 377C1 (5).

Domain	Criteria from the KCE NEED framework	Mentioned in literature sample on paediatric patients^a^	Findings from literature review	Relevance to paediatric population^b^
Patient UHNs				
Health	**Impact on general health-related quality of life (HRQoL)** *The extent to which a health condition impacts the general health-related quality of life of patients.*	✓		Generic
	**Impact on physical health** *The extent to which a health condition impacts the physical health of patients.*	Not/ Occasionally Reported but Relevant	Generic	
	**Impact on psychological health** *The extent to which a health condition psychological health of patients.*	✓	Widely reported, especially anxiety, depression, and emotional distress.	Generic
	**Impact on autonomy** *The extent to which a health condition impacts the autonomy of patients.*	✓	AYAs reported disrupted autonomy due to illness and treatment.	Generic
	**Impact on life expectancy** *The impact of a health condition on life expectancy of patients.*	Relevant but not systematically captured		Generic
Healthcare	**Effectiveness of treatment** *The extent to which the current overall treatment is effective.*	Relevant but not systematically captured		Generic
	**Burden of treatment** *The extent to which the current overall treatment is burdensome for patients.*	✓	Treatment was described as highly intensive, posing significant adherence challenges, particularly among AYAs.	Generic
	**Quality of care** *The extent to which the care provided, including its organisation, provision of information, involvement in decisions, and diagnostic processes, meets quality standard.*	✓	Age-appropriate information and involvement were frequently highlighted in the literature, emphasizing the need for tailored communication strategies and active participation of paediatric patients in their care, according to their developmental stage and maturity.	Generic
	**Accessibility of care** *The extent to which treatment is available and ease with which patients have access to the care they need.*	✓	Foregone care, particularly in relation to dental care, was frequently mentioned, as the management of the disease often takes precedence, leading to neglected dental health.	Generic
Social	**Impact on social life** *The impact of the health condition on patients’ social life, including on their social support needs and their capacity to establish meaningful relationships with the community, family and/or friends.*	✓	The impact on social relationships was frequently reported across all age groups, with AYAs particularly highlighting concerns about isolation, disrupted peer relationships, and fear regarding future prospects, including career opportunities, romantic relationships, and long-term social integration.	Paediatric-Adapted
	**Impact on education** *The impact of the health condition on patients’ education.*	✓	Unique to paediatric populations; treatment disrupts school attendance.	Paediatric-Specific
	**Impact on work** *The impact of the health condition on patients’ working conditions, including capacity to work, working hours and working environment.*	Not applicable		Non-paediatric
	**Financial consequences** *Costs borne by patients to be able to access reimbursed or non-reimbursed healthcare.*	Not Applicable^c^		Non-paediatric
Society UHNs				
Health	**Frequency** *How common a health condition is with reference to the size of the population (the population at risk) and a measure of time.*	Relevant but not systematically captured		Generic
	**Transmissibility** *The extent to which a health condition can be transmitted from one organism to another.*	Relevant but not systematically captured		Generic
	**Antimicrobial resistance** *The extent to which, due to drug resistance, antibiotics or other antimicrobial medicines used to treat or manage a health condition are ineffective, or infections related to a health condition become increasingly difficult or impossible to treat.*	Relevant but not systematically captured		Generic
	**Burden on informal caregivers** *The extent to which the quality of life of informal caregivers is affected by their caregiving activities.*	✓	The impact on the family as a whole was reported throughout literature, not limited to the parents. Also siblings experience an impact of the ill sibling. Needs related to mental health, information, family stability and organisation of care must be considered.	Paediatric-Adapted
Healthcare	**Value for money of standard of care** *The health gain achieved for the level of healthcare spending associated with the standard of care for a health condition. It allows for the identification of inefficiencies in the healthcare system.*	Relevant but not systematically captured		Generic
	**Preventability** *The extent to which a health condition can be avoided or mitigated through primary prevention strategies.*	Relevant but not systematically captured		Generic
Social	**Productivity losses** *The costs related to work absenteeism, presenteeism, early labour force exits of patients with the health condition and their caregivers.*	Relevant but not systematically captured		Generic
	**Environmental impact of standard of care** *The impact of the management of a health condition on the natural environment.*	Relevant but not systematically captured		Generic
Future UHNs				
Health	**The future burden of disease** *The impact of a health condition on the health of future generations.*	Relevant but not systematically captured		Generic
Healthcare and social	**Future economic burden** *The impact of a health condition on future healthcare expenditures and productivity, in monetary terms.*	Relevant but not systematically captured		Generic

a The label “Relevant but not systematically captured” indicates criteria deemed relevant for paediatric populations, but for which limited reporting was identified, possibly due to scope or focus limitations of existing studies.

b Paediatric-Specific = Criteria uniquely relevant or more pressing in paediatric populations. Paediatric-Adapted = Criteria relevant to both adults and children but requiring paediatric-specific adaptations. Generic = Criteria equally relevant to paediatric and adult populations without the need for age-specific adjustments. AYA, adolescents and yong adults; UHNs, unmet health-related needs.

c Some criteria are age-specific and might be considered relevant for adolescents and young adults (AYAs), as this group often resembles the adult population more closely. However, in this table, relevance was assessed with a focus on paediatric patients overall, without distinguishing specific applicability to AYAs.

## 4 Discussion

This review identified the age-specific UHNs of paediatric haematologic malignancies patients and their families, extending beyond medicinal or therapeutic gaps covered by UMN currently used in regulatory context, categorised across health, healthcare, and social domains. For paediatric patients with haematologic malignancies, five key criteria were identified: mental health, autonomy, forgone care, social interactions, and educational continuity. Among AYAs, additional needs emerged, including age-dependent information and involvement, treatment adherence, and future perspectives. For families, information needs were universally reported. Among informal caregivers, further challenges included mental health, privacy, organization of care, family stability, social interactions, and financial stability. Siblings specifically highlighted reduced parental attention as a significant UHN.

These findings indicate that children with haematologic malignancies and their families face a wide range of UHNs that extend beyond medical treatment and often stay unaddressed. Previous research aligns with our findings, as AYAs undergoing haematopoietic stem cell transplantation have greater information and support needs related to psychological health, body image, and sexuality than adults, and they also face UHNs concerning peer relationships, education, and fertility (7–9, 59–62). In contrast, less research exists on younger children, though depression and anxiety affect 6%–15% of paediatric patients (59). The study by Kahn et al. found that approximately 25% of patients demonstrated non-adherence during oral 6-mercaptopurine treatment (63). Notably, the majority of respondents were fathers or male caregivers, and 77% of the children with acute lymphoblastic leukemia (ALL) were older than 12 years. It is important to note that adherence in this study, as well as the studies we included in our review, was measured through self-report, which likely led to an underestimation of the true prevalence of non-adherence. Furthermore, the study reported a significant association between treatment adherence and parental education level, underscoring the relevance of socio-demographic factors in adherence behavior (63). Our findings regarding UHNs of informal caregivers aligned with previous findings, especially on the importance of organisation of care, which enhances their relationship with HCPs (52).

Although the present review focused on UHNs in haematologic malignancies, our findings align with literature regarding general paediatric oncology and even in the broader paediatric setting. Specific commonalities include mental health needs, the lack of being involved in investigations and decision-making, educational discontinuity, and reduced medication adherence (26, 52, 64–66). Specifically, social isolation and mental health unmet needs can be linked to a lack of normalcy during cancer care. To address this, clinicians should pay attention to restoring a sense of normalcy and support AYA quality of life by acknowledging and reinforcing identities that extend beyond the cancer diagnosis (67). Moreover, Mack et al. highlighted that 80% of more than 200 newly diagnosed AYAs with cancer considered it important to receive correct information on their prognosis (68). In line with our results, it was described that receiving more information resulted in greater trust in the HCP and less distress (68). Additionally, in a study from Rosenberg et al, investigating 35 AYAs with cancer with an average age of 17.6 years, it was found that sexual intimacy and substance use are common among AYAs. In this study, 40% of participants reported at least 1 communication need, including fertility, safe sex and birth control (69).The challenge of forgone care is also recognized by the European Commission, which specifically identifies dental care as an UHN (6). Finally, social determinants such as household composition and income influence the distribution and severity of unmet needs, reinforcing the importance of equity-sensitive assessment approaches. Additionally, children's UHNs vary based on household composition, with higher reported needs in single-parent households and lower-income families (6). Financial stability is a well-documented UHN among parents of children with cancer, which has been consistently associated with poorer parental emotional health and reduced quality of life (70–72). Parental work disruption is widespread, regardless of income level, while treatment-related costs that fall outside of insurance or universal healthcare coverage can cause significant additional expenses (72–74). Parental divorce or separation during or after treatment was not explicitly captured in the included studies, although it may represent a critical yet under-reported determinant of family stability and paediatric patients’ health-related needs.

Extrapolation of the identified UHN to other paediatric populations should be approached with caution. In line with the generic NEED framework, the application of UHN criteria requires a prior assessment of eligibility and relevance within each disease context. The relevance and expression of individual UHN criteria may therefore differ across paediatric chronic or life-threatening conditions and cannot be assumed *a priori*. Accordingly, extrapolation beyond haematological malignancies should be regarded as hypothesis-generating rather than confirmatory, and the framework should be applied as a flexible analytic tool following a structured relevance assessment. A similar sensitivity consideration applies to age categorisation, as the identified UHN criteria may differ if adolescents and young adults were excluded or if age-stratified analyses were available, reflecting developmental differences across paediatric age groups. Moreover, children with solid tumours might encounter distinct or more pronounced UHN, due to tumour locations and treatments. For example, as described in literature, children suffering from central nervous brain tumours might encounter neurological, cognitive and neuropsychological deficits (75, 76). Likewise, extremity sarcomas requiring amputation can generate functional impairments, and attention must be drawn to body image concerns, as these may persist long after treatment (77–79).

One of the key needs identified in this study by both children and their families is age-appropriate information and active involvement in care. A widely recognized concept in clinical practice is shared decision-making, which empowers patients to participate in their healthcare journey, however very frequently still lacking (80–82). This approach is often supported by digital tools, providing accessible information and facilitating informed decision-making. Approximately 60% of children aged between 0 and 11 years old, and more than 80% of AYAs have access to a smartphone or computer (83, 84). In this light, digital tools could be valuable as they have the potential to address UHNs in both children as well as their families. Several studies investigate the context in which these tools can be used, comprising information needs, medication adherence, and engagement in research, and whether paediatric patients and their families are receptive to them (59, 85–87).

This study can complement existing research initiatives that map health-related needs in the general population by adapting these frameworks for the paediatric setting, thus shaping decision-making and healthcare innovation towards a more patient-centred setting. Specifically, for the KCE NEED framework, the inclusion of paediatric-specific criteria, such as age-dependent information and involvement, future perspectives, and treatment adherence, could be considered. Additionally, the broader impact on families, including siblings, could be integrated into such assessments to provide a comprehensive understanding of paediatric healthcare needs. Lastly, developmental and age-stratified dimensions should be incorporated to capture differences across childhood, adolescence, and young adulthood.

It is important to clarify the relationship between the UHN identified in this study and the concept of UMN as applied in regulatory and policy contexts. UMN is most commonly operationalized with a primary focus on therapeutic or medicinal gaps. In contrast, the findings of this review demonstrate that paediatric patients and their families experience a broader and interconnected set of health-related needs that extend beyond medical treatment alone. Accordingly, the NEED framework applied in this study is not intended to redefine UMN, but rather to offer a structured and transparent approach to identifying additional dimensions of need, including psychosocial, developmental, family-level, and system-related aspects, which may, where considered relevant by decision-makers, inform UMN assessments.

Recent years have seen a gradual shift in many healthcare policy discussions toward a more holistic understanding of patient needs, including psychological support, information provision, and shared decision-making, while medicine-oriented approaches remain central. Concurrently, the 2023 proposal for a reform of the pharmaceutical legislation highlights the concept of unmet medical needs, aiming to steer R&D toward areas with significant gaps, such as paediatric diseases (88). While this growing focus is promising, it is essential to adopt a holistic approach that extends beyond medicinal needs. Based on this study's findings, it is recommended that other health-related impacts, such as psychosocial support, educational continuity, and family well-being, are considered in decisions related to reimbursement policies, research priorities, and treatment development.

### 4.1 Limitations and future research

The generalisability of this review to the broader paediatric population (0–18 years, across all diseases) is subject to several limitations related to the methodological characteristics of the included studies. First, many included studies relied on parent-proxy investigation for patient UHN or relatively small samples, which may limit the representativeness of the findings. Second, heterogeneity in how “paediatric” populations were defined, particularly when adolescents and young adults (AYAs, 12–24 years) were included, complicated cross-study comparison. Third, unmet needs vary substantially across developmental stages, yet much of the available literature focused on older children and adolescents, resulting in underrepresentation of infants and younger children. Additional methodological challenges included the frequent use of cross-sectional study designs, limiting insight into how unmet needs evolve over the disease trajectory, as well as variation in consent procedures and data collection approaches, reflecting the ethical and practical complexities of conducting research in paediatric populations. These limitations may contribute to an overrepresentation of needs that are more readily observable or reportable by parents or adolescents, while needs relevant to younger children or emerging over time may be less visible. Although this review distinguished between two age categories where differences appeared most pronounced, these age bands may not capture the full complexity of developmental trajectories.

These findings underscore the need for future primary research employing longitudinal designs, age-stratified analyses, and mixed-methods approaches that integrate child, caregiver, and clinician perspectives. Greater methodological consistency and explicit reporting of age-related and developmental considerations would strengthen the evidence base for identifying unmet health-related needs in paediatric populations and support more robust application of needs-based frameworks.

Finally, the review centred on children with haematological malignancies. This focus was chosen because these conditions span the full paediatric age range and are among the most prevalent childhood cancers, which supports cautious extrapolation. However, any extrapolation should be considered hypothesis-generating, followed by empirical validation in the specific paediatric condition. For example, solid tumours, as described earlier, may result in additional or more amplified UHN.

The classification of certain criteria as “relevant but not systematically captured” likely reflects differences in study scope, methodology, and level of analysis rather than a lack of conceptual relevance. First, criteria such as impact on life expectancy and effectiveness of treatment are typically addressed through clinical trials, registries, or epidemiological studies, whereas the present review focused on needs-based assessments that prioritise lived experiences of patients and families. As a result, these clinically oriented criteria fall largely outside the epistemological focus of the included studies. Second, methodological blind spots, such as the exclusion of studies focusing exclusively on long-term survivors, may have limited the capture of outcomes like productivity losses, thereby contributing to the underrepresentation of these criteria in the present review. Third, societal-level NEED criteria, including frequency, transmissibility, antimicrobial resistance, preventability, productivity losses, environmental impact of care, and value for money of standard of care, were rarely addressed. This pattern suggests methodological blind spots in paediatric needs research, where broader population-level and economic considerations are seldom integrated into paediatric needs assessments, rather than fundamental differences in the relevance of these criteria for paediatric populations.

This review highlights five areas where future research is essential: (i) As most existing studies primarily address AYAs, future research should focus on the specific UHNs of younger children. (ii) Qualitative methods, such as interviews and focus groups, would provide deeper insights and support the integration of findings into frameworks like the NEED framework. (iii) Long-term effects on survivors require further exploration. While survival rates are high, intensive treatments may lead to co-morbidities and reduced quality of life, emphasizing the need for longitudinal studies assessing outcomes into adulthood (89). (iv) Research on foregone care, such as dental care, is also necessary to understand missed healthcare services and their long-term impact. (v) Finally, social determinants of health remain underexplored. Factors such as household composition and income significantly affect UHNs, with single-parent and lower-income households reporting higher needs (6). Addressing these factors is crucial for equitable paediatric oncology care.

## 5 Conclusion

This study highlights the need for a holistic approach to address the UHNs of paediatric haematologic malignancies patients and their families, extending beyond medical treatment to include age-specific information, treatment adherence, and mental health support. Existing frameworks, such as the KCE NEED framework, should be tailored for application in the paediatric population by incorporating these paediatric-specific criteria. The increasing focus on unmet medical needs in European health policy is a unique opportunity to ensure that research priorities, reimbursement decisions, and treatment development consider broader health impacts, promoting comprehensive, patient-centred paediatric care. From a practice and policy perspective, this study shows the importance of routine implementation of identified aspects, such as providing age-appropriate information and involvement of paediatric patients and their needs in clinical and regulatory decision-making. Lastly, family criteria should be included in needs assessment, to capture family mental health, sibling outcomes, and socioeconomic strains.

## Data Availability

The original contributions presented in the study are included in the article/Supplementary Material, further inquiries can be directed to the corresponding author.
