# Supplementary material for: Criteria for unmet need in paediatric populations and their families: a literature-based case study in haematological malignancies in upper-middle and high-income countries
Source: Front Pediatr. 2026 Feb 13;14:1765938. doi: 10.3389/fped.2026.1765938 (PMC12946065; doi:10.3389/fped.2026.1765938)
Supplement: Supplementary file 1 [file Datasheet1.pdf]

Criteria for unmet need in paediatric populations and their families: a literature-based case study in haematological malignancies in upper-middle and high-income countries

Charlotte Van Isterdael[1,†], Zilke Claessens[1,2,†], Isabelle Huys[1]

[1]Department of Pharmaceutical and Pharmacological Sciences, KU Leuven, Leuven, Belgium

[2]Research Foundation Flanders, Brussel, Belgium

[†]These authors share first authorship

Supplementary Information I: search string of scoping literature review

Concept 1: methods

Concept 2: unmet needs

Concept 3: paediatrics

Concept 4: leukaemia/lymphoma

→ search string: (concept 1) AND (concept 2) AND (concept 3) AND (concept 4)

Pubmed

(“Surveys and Questionnaires"[Mesh] OR “survey*"[Tiab] OR “questionnaire*"[Tiab] OR “self report”[Tiab:~3] OR “self reports”[Tiab:~3] OR “self reported”[Tiab:~3] OR “self reporting”[Tiab:~3] OR “Self Report”[Mesh] OR “Qualitative Research"[Mesh] OR “Qualitative research”[Tiab:~3] OR “Qualitative study”[Tiab:~3] OR “Qualitative studies”[Tiab:~3] OR “Quantitative research”[Tiab:~3] OR “Quantitative study”[Tiab:~3] OR “Quantitative studies”[Tiab:~3] OR “Interviews as Topic"[Mesh] OR “Interview*"[Tiab] OR “Focus Groups"[Mesh] OR “Focus group*"[Tiab] OR “Focus discussion*"[Tiab] OR “Multi criteria decision analysis"[Tiab] OR “Multiple criteria decision analysis"[Tiab] OR “Multicriteria decision analysis"[Tiab] OR “Multi criteria decision analyses"[Tiab] OR “Multiple criteria decision analyses"[Tiab] OR “Multicriteria decision analyses"[Tiab] OR “Multi criteria decision making"[Tiab] OR “Multiple criteria decision making"[Tiab] OR “Multicriteria decision making"[Tiab] OR “Multi criteria decision tool*"[Tiab] OR “MCDA"[Tiab] OR “Registries"[Mesh] OR “Registries"[Tiab] OR “Registry"[Tiab] OR “Register*"[Tiab] OR “Workshop*"[Tiab] OR “Patient Participation"[Mesh] OR “patient empowerment”[Tiab:~6] OR “patients empowerment”[Tiab:~6] OR “engagement method"[Tiab:~6] OR “engagement methods"[Tiab:~6] OR “Needs Assessment"[Mesh] OR “Patient Outcome Assessment"[Mesh] OR “visual analogue scale*”[Tiab] OR “EQ-5D”[Tiab] OR “Health Services Research"[Mesh] OR “expert panel”[Tiab:~6] OR “expert panels”[Tiab:~6] OR “experts panels”[Tiab:~6] OR “experts panel”[Tiab:~6] OR “expert forum"[Tiab:~6] OR “expert forums"[Tiab:~6] OR “experts forums"[Tiab:~6] OR “experts forum"[Tiab:~6] OR “expert consultation"[Tiab:~6] OR

“expert consultations"[Tiab:~6] OR “experts consultations"[Tiab:~6] OR “experts consultation"[Tiab:~6] OR “consulting expert"[Tiab:~6] OR “consulting experts"[Tiab:~6] OR “expert participation"[Tiab:~6] OR “expert participations"[Tiab:~6] OR “experts participation"[Tiab:~6] OR “experts participation"[Tiab:~6] OR “participating expert"[Tiab:~6] OR “participating experts"[Tiab:~6] OR “expert involvement"[Tiab:~6] OR “experts involvements"[Tiab:~6] OR “experts involvement"[Tiab:~6] OR “expert involvements"[Tiab:~6] OR “involving expert"[Tiab:~6] OR “involving experts"[Tiab:~6] OR “expert engagement"[Tiab:~6] OR “experts engagement"[Tiab:~6] OR “expert engagements"[Tiab:~6] OR “experts engagements"[Tiab:~6] OR “engaging expert"[Tiab:~6] OR “engaging experts"[Tiab:~6] OR “expert network"[Tiab:~6] OR “expert networks"[Tiab:~6] OR “experts network"[Tiab:~6] OR “experts networks"[Tiab:~6] OR “expert networking"[Tiab:~6] OR “experts networking"[Tiab:~6] OR “social network"[Tiab:~6] OR “social networks"[Tiab:~6] OR “social networking"[Tiab:~6] OR “patient panel”[Tiab:~6] OR “patient panels”[Tiab:~6] OR “patients panels”[Tiab:~6] OR “patients panel”[Tiab:~6] OR “patient forum"[Tiab:~6] OR “patient forums"[Tiab:~6] OR “patients forums"[Tiab:~6] OR “patients forum"[Tiab:~6] OR “patient consultation"[Tiab:~6] OR “patient consultations"[Tiab:~6] OR “patients consultations"[Tiab:~6] OR “patients consultation"[Tiab:~6] OR “consulting patient"[Tiab:~6] OR “consulting patients"[Tiab:~6] OR “patient participation"[Tiab:~6] OR “patient participations"[Tiab:~6] OR “patients participation"[Tiab:~6] OR “patients participation"[Tiab:~6] OR “participating patient"[Tiab:~6] OR “participating patients"[Tiab:~6] OR “patient involvement"[Tiab:~6] OR “patients involvements"[Tiab:~6] OR “patients involvement"[Tiab:~6] OR “patient involvements"[Tiab:~6] OR “involving patient"[Tiab:~6] OR “involving patients"[Tiab:~6] OR “patient engagement"[Tiab:~6] OR “patients engagement"[Tiab:~6] OR “patient engagements"[Tiab:~6] OR “patients engagements"[Tiab:~6] OR “engaging patient"[Tiab:~6] OR “engaging patients"[Tiab:~6] OR “patient network"[Tiab:~6] OR “patient networks"[Tiab:~6] OR “patients network"[Tiab:~6] OR “patients networks"[Tiab:~6] OR “patient networking"[Tiab:~6] OR “patients networking"[Tiab:~6] OR “patient activation"[Tiab:~6] OR “patient activations"[Tiab:~6] OR “patients activation"[Tiab:~6] OR “patients activations"[Tiab:~6] OR “activating patient"[Tiab:~6] OR “activating patient"[Tiab:~6] OR “stakeholder panel”[Tiab:~6] OR “stakeholder panels”[Tiab:~6] OR “stakeholders panels”[Tiab:~6] OR “stakeholders panel”[Tiab:~6] OR “stakeholder forum"[Tiab:~6] OR “stakeholder forums"[Tiab:~6] OR “stakeholders forums"[Tiab:~6] OR “stakeholders forum"[Tiab:~6] OR “stakeholder consultation"[Tiab:~6] OR “stakeholder consultations"[Tiab:~6] OR “stakeholders consultations"[Tiab:~6] OR “stakeholders consultation"[Tiab:~6] OR “consulting stakeholder"[Tiab:~6] OR “consulting stakeholders"[Tiab:~6] OR “stakeholder participation"[Tiab:~6] OR “stakeholder participations"[Tiab:~6] OR “stakeholders participation"[Tiab:~6] OR “stakeholders participation"[Tiab:~6] OR “participating stakeholder"[Tiab:~6] OR “participating stakeholders"[Tiab:~6] OR “stakeholder involvement"[Tiab:~6] OR “stakeholders involvements"[Tiab:~6] OR “stakeholders

involvement"[Tiab:~6] OR “stakeholder involvements"[Tiab:~6] OR “involving stakeholder"[Tiab:~6] OR “involving stakeholders"[Tiab:~6] OR “stakeholder engagement"[Tiab:~6] OR “stakeholders engagement"[Tiab:~6] OR “stakeholder engagements"[Tiab:~6] OR “stakeholders engagements"[Tiab:~6] OR “engaging stakeholder"[Tiab:~6] OR “engaging stakeholders"[Tiab:~6] OR “stakeholder network"[Tiab:~6] OR “stakeholder networks"[Tiab:~6] OR “stakeholders network"[Tiab:~6] OR “stakeholders networks"[Tiab:~6] OR “stakeholder networking"[Tiab:~6] OR “stakeholders networking"[Tiab:~6] OR “stakeholder activation"[Tiab:~6] OR “stakeholder activations"[Tiab:~6] OR “stakeholders activation"[Tiab:~6] OR “stakeholders activations"[Tiab:~6] OR “activating stakeholder"[Tiab:~6] OR “activating stakeholder"[Tiab:~6] OR “society panel”[Tiab:~6] OR “society panels”[Tiab:~6] OR “society forum"[Tiab:~6] OR “society forums"[Tiab:~6] OR “society consultation"[Tiab:~6] OR “society consultations"[Tiab:~6] OR “society consulting"[Tiab:~6] OR “society participation"[Tiab:~6] OR “society participations"[Tiab:~6] OR “society participating"[Tiab:~6] OR “society involvement"[Tiab:~6] OR “society involvements"[Tiab:~6] OR “society involving"[Tiab:~6] OR “society engagement"[Tiab:~6] OR “society engagements"[Tiab:~6] OR “society engaging"[Tiab:~6] OR “society network"[Tiab:~6] OR “society networks"[Tiab:~6] OR “society activation"[Tiab:~6] OR “society activations"[Tiab:~6] OR “society activating"[Tiab:~6] OR “needs assessment”[Tiab:~6] OR “need assessment”[tiab:~6] OR “needs assessments”[Tiab:~6] OR “need assessments”[tiab:~6]OR “assessing need”[tiab:~6] OR “assessing needs”[Tiab:~6] OR “need determination”[Tiab:~6] OR "determining need”[Tiab:~6] OR “needs determination”[Tiab:~6] OR “determining needs”[Tiab:~6] OR “need determinations”[Tiab:~6] OR “needs determinations”[Tiab:~6] OR “patient outcome*”[Tiab] OR “patients outcome*”[Tiab] OR “epidemiologic research"[Tiab:~6] OR “epidemiological research"[Tiab:~6] OR “epidemiologic method"[Tiab:~6] OR “epidemiological methods"[Tiab:~6] OR “epidemiologic study"[Tiab:~6] OR “epidemiological study"[Tiab:~6] OR “epidemiologic studies"[Tiab:~6] OR “epidemiological studies"[Tiab:~6] OR “health services research”[Tiab:~6] OR “health service research”[Tiab:~6] OR “health services evaluation”[Tiab:~6] OR “health services evaluations”[Tiab:~6] OR “health service evaluation”[Tiab:~6] OR “health service evaluations”[Tiab:~6] OR “health care research”[Tiab:~6] OR “healthcare research”[Tiab:~6] OR “observational research”[Tiab:~6] OR “Observational studies”[Tiab:~6] OR “Observational study”[Tiab:~6] OR “cohort research”[Tiab:~6] OR “cohort study”[Tiab:~6] OR “cohort studies”[Tiab:~6] OR “case-control study”[Tiab:~6] OR “case-control studies”[Tiab:~6] OR “healthcare evaluation”[Tiab:~6] OR “health care evaluation”[Tiab:~6] OR “health-care evaluation”[Tiab:~6])

AND

("unmet need"[Tiab:~6] OR “unmet needs"[Tiab:~6] OR “unmet demand"[Tiab:~6] OR “unmet demands"[Tiab:~6] OR “societal need"[Tiab:~6] OR “societal needs"[Tiab:~6] OR “societal demand"[Tiab:~6] OR “societal demands"[Tiab:~6] OR “society need"[Tiab:~6] OR “society needs"[Tiab:~6] OR “society demand"[Tiab:~6] OR “society demands"[Tiab:~6] OR “community need"[Tiab:~6] OR “community needs"[Tiab:~6] OR “community demand"[Tiab:~6] OR “community demands"[Tiab:~6] OR “communities need"[Tiab:~6] OR “communities needs"[Tiab:~6] OR “communities demand"[Tiab:~6] OR “communities demands"[Tiab:~6] OR “health need"[Tiab:~6] OR “health needs"[Tiab:~6] OR “health demand"[Tiab:~6] OR “health demands"[Tiab:~6] OR “healthcare need"[Tiab:~6] OR “healthcare needs"[Tiab:~6] OR “Healthcare demand"[Tiab:~6] OR “Healthcare demands"[Tiab:~6] OR “health care need"[Tiab:~6] OR “health care needs"[Tiab:~6] OR “Health care demand"[Tiab:~6] OR “Health care demands"[Tiab:~6] OR “care need”[Tiab:~6] OR “care needs"[Tiab:~6] OR “care demand”[Tiab:~6] OR “care demands”[Tiab:~6] OR “medical need"[Tiab:~6] OR “medical needs"[Tiab:~6] OR “medical demand"[Tiab:~6] OR “medical demands"[Tiab:~6] OR “therapeutic need"[Tiab:~6] OR “therapeutic needs"[Tiab:~6] OR “therapeutic demand"[Tiab:~6] OR “therapeutic demands"[Tiab:~6] OR “therapeutical need"[Tiab:~6] OR “therapeutical needs"[Tiab:~6] OR “therapeutical demand"[Tiab:~6] OR “therapeutical demands"[Tiab:~6] OR “pharmaceutic need"[Tiab:~6] OR “pharmaceutic needs"[Tiab:~6] OR “pharmaceutic demand"[Tiab:~6] OR “pharmaceutic demands"[Tiab:~6] OR “pharmaceutical need"[Tiab:~6] OR “pharmaceutical needs"[Tiab:~6] OR “pharmaceutical demand"[Tiab:~6] OR “pharmaceutical demands"[Tiab:~6] OR “patient need"[Tiab:~6] OR “patient needs"[Tiab:~6] OR “patient demand"[Tiab:~6] OR “patient demands"[Tiab:~6] OR “patients need"[Tiab:~6] OR “patients needs"[Tiab:~6] OR “patients demand"[Tiab:~6] OR “patients demands"[Tiab:~6] OR “population need"[Tiab:~6] OR “population needs"[Tiab:~6] OR “population demand"[Tiab:~6] OR “population demands"[Tiab:~6] OR “populations need"[Tiab:~6] OR “populations needs"[Tiab:~6] OR “populations demand"[Tiab:~6] OR “populations demands"[Tiab:~6] OR “therapy need"[Tiab:~6] OR “therapy needs"[Tiab:~6] OR “therapy demand"[Tiab:~6] OR “therapy demands"[Tiab:~6])

AND

(“Pediatrics"[Mesh] OR “Pediatric*"[Tiab] OR “Paediatric*"[Tiab] OR “Child"[Mesh] OR “Child*"[Tiab] OR “Child, Hospitalized"[Mesh] OR “Toddler*"[Tiab] OR “Infant"[Mesh] OR “Infan*" [Tiab] OR “Adolescent, Hospitalized"[Mesh] OR “Newborn*" [Tiab] OR “neonat*"[Tiab] OR “perinat*"[Tiab] OR “Minors"[Mesh] OR “Minors"[Tiab] OR “Adolescent"[Mesh] OR “Adolescen*"[Tiab] OR “Teen*"[Tiab] OR “Youth*"[Tiab] OR (("1 week"[tiab] OR "2 week*"[tiab] OR "3 week*"[tiab] OR "4 week*"[tiab] OR "5 week*"[tiab] OR "6 week*"[tiab] OR "7 week*"[tiab] OR "8 week*"[tiab] OR "9 week*"[tiab] OR "10

week*"[tiab] OR "11 week*"[tiab] OR "12 week*"[tiab] OR "13 week*"[tiab] OR "14 week*"[tiab] OR "15 week*"[tiab] OR "16 week*"[tiab] OR "one week"[tiab] OR "two week*"[tiab] OR "three week*"[tiab] OR "four week*"[tiab] OR "five week*"[tiab] OR "six week*"[tiab] OR "seven week*"[tiab] OR "eight week*"[tiab] OR "nine week*"[tiab] OR "ten week*"[tiab] OR "eleven week*"[tiab] OR "twelve week*"[tiab] OR "thirteen week*"[tiab] OR "fourteen week*"[tiab] OR "fifteen week*"[tiab] OR "sixteen week*"[tiab] OR "17 week*"[tiab] OR "18 week*"[tiab] OR "19 week*"[tiab] OR "20 week*"[tiab] OR "21 week*"[tiab] OR "22 week*"[tiab] OR "23 week*"[tiab] OR "24 week*"[tiab] OR "25 week*"[tiab] OR "26 week*"[tiab] OR "27 week*"[tiab] OR "28 week*"[tiab] OR "29 week*"[tiab] OR "30 week*"[tiab] OR "31 week*"[tiab] OR "32 week*"[tiab] OR "1 month"[tiab] OR "2 month*"[tiab] OR "3 month*"[tiab] OR "4 month*"[tiab] OR "5 month*"[tiab] OR "6 month*"[tiab] OR "7 month*"[tiab] OR "8 month*"[tiab] OR "9 month*"[tiab] OR "10 month*"[tiab] OR "11 month*"[tiab] OR "12 month*"[tiab] OR "13 month*"[tiab] OR "14 month*"[tiab] OR "15 month*"[tiab] OR "16 month*"[tiab] OR "one month"[tiab] OR "two month*"[tiab] OR "three month*"[tiab] OR "four month*"[tiab] OR "five month*"[tiab] OR "six month*"[tiab] OR "seven month*"[tiab] OR "eight month*"[tiab] OR "nine month*"[tiab] OR "ten month*"[tiab] OR "eleven month*"[tiab] OR "twelve month*"[tiab] OR "thirteen month*"[tiab] OR "fourteen month*"[tiab] OR "fifteen month*"[tiab] OR "sixteen month*"[tiab] OR "17 month*"[tiab] OR "18 month*"[tiab] OR "19 month*"[tiab] OR "20 month*"[tiab] OR "21 month*"[tiab] OR "22 month*"[tiab] OR "23 month*"[tiab] OR "24 month*"[tiab] OR "1 year"[tiab] OR "2 year*"[tiab] OR "2 year*"[tiab] OR "3 year*"[tiab] OR "4 year*"[tiab] OR "5 year*"[tiab] OR "6 year*"[tiab] OR "7 year*"[tiab] OR "8 year*"[tiab] OR "9 year*"[tiab] OR "10 year*"[tiab] OR "11 year*"[tiab] OR "12 year*"[tiab] OR "13 year*"[tiab] OR "14 year*"[tiab] OR "15 year*"[tiab] OR "16 year*"[tiab] OR "one year"[tiab] OR "two year*"[tiab] OR "three year*"[tiab] OR "3 year*"[tiab] OR "four year*"[tiab] OR "five year*"[tiab] OR "six year*"[tiab] OR "seven year*"[tiab] OR "eight year*"[tiab] OR "nine year*"[tiab] OR "ten year*"[tiab] OR "eleven year*"[tiab] OR "twelve year*"[tiab] OR "thirteen year*"[tiab] OR "fourteen year*"[tiab] OR "fifteen year*"[tiab] OR "sixteen year*"[tiab] OR "17 year*"[tiab] OR "18 year*"[tiab]) AND ("old"[tiab] OR "age"[tiab] OR "aged"[tiab] OR "ages"[tiab])) OR "day old"[Tiab] OR "days old"[Tiab] OR "month old"[Tiab] OR "months old"[Tiab] OR "juvenile*"[Tiab] OR "kid"[Tiab] OR "kids"[Tiab] OR "menarche"[Tiab] OR "puberty"[mesh] OR "prepuber*"[Tiab] OR "puber*"[Tiab] OR "school age*"[Tiab] OR "schoolchild*"[Tiab] OR "preschool"[tiab] OR "preteen*"[Tiab] OR "teen*"[Tiab] OR "youngster*"[Tiab] OR "first grade*"[Tiab] OR "second grade*"[Tiab] OR "third grade*"[Tiab] OR "fourth grade*"[Tiab] OR "fifth grade*"[Tiab] OR "sixth grade*"[Tiab] OR "seventh grade*"[Tiab] OR "eight grade*"[Tiab] OR "ninth grade*"[Tiab] OR "tenth grade*"[Tiab] OR "eleventh grade*"[Tiab] OR "twelfth grade*"[Tiab] OR "grade 1"[Tiab] OR "grade 2"[Tiab] OR "grade 3"[Tiab] OR "grade 4"[Tiab] OR "grade 5"[Tiab] OR "grade 6"[Tiab] OR "grade 7"[Tiab] OR "grade 8"[Tiab] OR

“grade 9”[Tiab] OR “grade 10”[Tiab] OR “grade 11”[Tiab] OR “grade 12”[Tiab] OR “grade one”[Tiab] OR “grade two”[Tiab] OR “grade three”[Tiab] OR “grade four”[Tiab] OR “grade five”[Tiab] OR “grade six”[Tiab] OR “grade seven”[Tiab] OR “grade eight”[Tiab] OR “grade nine”[Tiab] OR “grade ten”[Tiab] OR “grade twelve”[Tiab] OR “low birthweight"[tiab] OR “birth"[tiab] OR “born"[tiab] OR “immatur*"[tiab] OR “NICU"[tiab] OR “postnat*"[tiab] OR “post-nat*"[tiab] OR “prematur*"[tiab] OR “pre-nat*"[tiab] OR “preterm"[tiab] OR “pre-term"[tiab] OR “LBW"[tiab] OR “VLBW"[tiab] OR “ELBW"[tiab] OR “VPT"[tiab] OR “nursery”[tiab] OR “childbirth”[tiab] OR “congenital”[tiab] OR “baby”[tiab] OR “babies”[tiab] OR "Infant, Low Birth Weight"[Mesh])

AND

(“leukemia”[mesh] OR “Leukemia*"[Tiab] OR “leukaemia*”[tiab] OR “AML”[Tiab] OR “MPAL*”[tiab] OR “APL”[tiab] OR “CML”[Tiab] OR “JMML”[Tiab] OR “Hematologic Neoplasms”[Mesh] OR “Leucocythemia*”[tiab] OR “Leucocythaemia*”[tiab] OR "Lymphoma"[Mesh] OR “lymphoma*”[tiab] OR “non-Hodgkin”[tiab] OR “Hodgkin”[tiab] OR “PMBL”[tiab] OR “HL”[tiab] OR “NSCHL”[tiab] OR “NLPHL”[tiab] OR “MCCHL”[tiab] OR “NHL”[tiab] OR “LBL”[tiab] OR “Burkitt”[tiab] OR “DLBCL”[tiab] OR “ALCL”[tiab] OR ((“blood”[tiab] OR “hematologic”[tiab] OR “haematologic”[tiab]) AND (“cancer*”[tiab] OR “tumor*”[tiab] OR “tumour*”[tiab] OR “neoplasm*”[tiab] OR “carcinoma*”[tiab])))

Embase

(‘questionnaire'/exp OR 'self report'/exp OR 'qualitative research'/exp OR 'quantitative study'/exp OR 'interview'/exp OR 'multicriteria decision analysis'/exp OR 'register'/exp OR 'workshop'/exp OR 'patient participation'/exp OR 'patient empowerment'/exp OR 'patient engagement'/exp OR 'social network'/exp OR 'needs assessment'/exp OR 'health services research'/exp OR ‘survey*’:ti,ab,kw OR ‘questionnaire*’:ti,ab,kw OR ((‘self’ NEAR/4 ‘report’):ti,ab,kw) OR ((‘self’ NEAR/4 ‘reports’):ti,ab,kw) OR ((‘self’ NEAR/4 ‘reported’):ti,ab,kw) OR ((‘self’ NEAR/4 ‘reporting’):ti,ab,kw) OR ((‘Qualitative’ NEAR/4 ‘research’):ti,ab,kw) OR ((‘Qualitative’ NEAR/4 ‘study’):ti,ab,kw) OR ((‘Qualitative’ NEAR/4 ‘studies’):ti,ab,kw) OR ((‘Quantitative’ NEAR/4 ‘research’):ti,ab,kw) OR ((‘Quantitative’ NEAR/4 ‘study’):ti,ab,kw) OR ((‘Quantitative’ NEAR/4 ‘studies’):ti,ab,kw) OR ‘Interview*’:ti,ab,kw OR ‘Focus group*’:ti,ab,kw OR ‘Focus discussion*’:ti,ab,kw OR ‘Multi criteria decision analysis’:ti,ab,kw OR ‘Multiple criteria decision analysis’:ti,ab,kw OR ‘Multicriteria decision analysis’:ti,ab,kw OR ‘Multi criteria decision analyses’:ti,ab,kw OR ‘Multiple criteria decision analyses’:ti,ab,kw OR ‘Multicriteria decision analyses’:ti,ab,kw OR ‘Multi criteria decision making’:ti,ab,kw OR ‘Multiple criteria decision

making’:ti,ab,kw OR ‘Multicriteria decision making’:ti,ab,kw OR ‘Multi criteria decision tool*’:ti,ab,kw OR ‘MCDA’:ti,ab,kw OR ‘Registries’:ti,ab,kw OR ‘Registry’:ti,ab,kw OR ‘Register*’:ti,ab,kw OR ‘Workshop*’:ti,ab,kw OR ((‘patient’ NEAR/7 ‘empowerment’):ti,ab,kw) OR ((‘patients’ NEAR/7 ‘empowerment’):ti,ab,kw) OR ((‘engagement’ NEAR/7 ‘method’):ti,ab,kw) OR ((‘engagement’ NEAR/7 ‘methods’):ti,ab,kw) OR ‘visual analogue scale*’:ti,ab,kw OR ‘EQ-5D’:ti,ab,kw OR (('expert' NEAR/7 'panel'):ti,ab,kw) OR (('expert' NEAR/7 'panels'):ti,ab,kw) OR (('experts' NEAR/7 'panels'):ti,ab,kw) OR (('experts' NEAR/7 'panel'):ti,ab,kw) OR (('expert' NEAR/7 'forum'):ti,ab,kw) OR (('expert' NEAR/7 'forums'):ti,ab,kw) OR (('experts' NEAR/7 'forums'):ti,ab,kw) OR (('experts' NEAR/7 'forum'):ti,ab,kw) OR (('expert' NEAR/7 'consultation'):ti,ab,kw) OR (('expert' NEAR/7 'consultations'):ti,ab,kw) OR (('experts' NEAR/7 'consultations'):ti,ab,kw) OR (('experts' NEAR/7 'consultation'):ti,ab,kw) OR (('consulting' NEAR/7 'expert'):ti,ab,kw) OR (('consulting' NEAR/7 'experts'):ti,ab,kw) OR (('expert' NEAR/7 'participation'):ti,ab,kw) OR (('expert' NEAR/7 'participations'):ti,ab,kw) OR (('experts' NEAR/7 'participation'):ti,ab,kw) OR 'experts participation':ti,ab,kw OR (('participating' NEAR/7 'expert'):ti,ab,kw) OR (('participating' NEAR/7 'experts'):ti,ab,kw) OR (('expert' NEAR/7 'involvement'):ti,ab,kw) OR (('experts' NEAR/7 'involvements'):ti,ab,kw) OR (('experts' NEAR/7 'involvement'):ti,ab,kw) OR (('expert' NEAR/7 'involvements'):ti,ab,kw) OR (('involving' NEAR/7 'expert'):ti,ab,kw) OR (('involving' NEAR/7 'experts'):ti,ab,kw) OR (('expert' NEAR/7 'engagement'):ti,ab,kw) OR (('experts' NEAR/7 'engagement'):ti,ab,kw) OR (('expert' NEAR/7 'engagements'):ti,ab,kw) OR (('experts' NEAR/7 'engagements'):ti,ab,kw) OR (('engaging' NEAR/7 'expert'):ti,ab,kw) OR (('engaging' NEAR/7 'experts'):ti,ab,kw) OR (('expert' NEAR/7 'network'):ti,ab,kw) OR (('expert' NEAR/7 'networks'):ti,ab,kw) OR (('experts' NEAR/7 'network'):ti,ab,kw) OR (('experts' NEAR/7 'networks'):ti,ab,kw) OR (('expert' NEAR/7 'networking'):ti,ab,kw) OR (('experts' NEAR/7 'networking'):ti,ab,kw) OR (('social' NEAR/7 'network'):ti,ab,kw) OR (('social' NEAR/7 'networks'):ti,ab,kw) OR ((‘social’ NEAR/7 ‘networking’):ti,ab,kw) OR 'patient panel':ti,ab,kw OR (('patient' NEAR/7 'panels'):ti,ab,kw) OR (('patients' NEAR/7 'panels'):ti,ab,kw) OR (('patients' NEAR/7 'panel'):ti,ab,kw) OR (('patient' NEAR/7 'forum'):ti,ab,kw) OR (('patient' NEAR/7 'forums'):ti,ab,kw) OR (('patients' NEAR/7 'forums'):ti,ab,kw) OR (('patients' NEAR/7 'forum'):ti,ab,kw) OR (('patient' NEAR/7 'consultation'):ti,ab,kw) OR (('patient' NEAR/7 'consultations'):ti,ab,kw) OR (('patients' NEAR/7 'consultations'):ti,ab,kw) OR (('patients' NEAR/7 'consultation'):ti,ab,kw) OR (('consulting' NEAR/7 'patient'):ti,ab,kw) OR (('consulting' NEAR/7 'patients'):ti,ab,kw) OR (('patient' NEAR/7 'participation'):ti,ab,kw) OR (('patient' NEAR/7 'participations'):ti,ab,kw) OR (('patients' NEAR/7 'participation'):ti,ab,kw) OR (('participating' NEAR/7 'patient'):ti,ab,kw) OR (('participating' NEAR/7 'patients'):ti,ab,kw) OR (('patient' NEAR/7 'involvement'):ti,ab,kw) OR (('patients' NEAR/7 'involvements'):ti,ab,kw) OR (('patients' NEAR/7 'involvement'):ti,ab,kw) OR (('patient' NEAR/7 'involvements'):ti,ab,kw) OR (('involving' NEAR/7

'patient'):ti,ab,kw) OR (('involving' NEAR/7 'patients'):ti,ab,kw) OR (('patient' NEAR/7 'engagement'):ti,ab,kw) OR (('patients' NEAR/7 'engagement'):ti,ab,kw) OR (('patient' NEAR/7 'engagements'):ti,ab,kw) OR (('patients' NEAR/7 'engagements'):ti,ab,kw) OR (('engaging' NEAR/7 'patient'):ti,ab,kw) OR (('engaging' NEAR/7 'patients'):ti,ab,kw) OR (('patient' NEAR/7 'network'):ti,ab,kw) OR (('patient' NEAR/7 'networks'):ti,ab,kw) OR (('patients' NEAR/7 'network'):ti,ab,kw) OR (('patients' NEAR/7 'networks'):ti,ab,kw) OR (('patient' NEAR/7 'networking'):ti,ab,kw) OR (('patients' NEAR/7 'networking'):ti,ab,kw) OR (('patient' NEAR/7 'activation'):ti,ab,kw) OR (('patient' NEAR/7 'activations'):ti,ab,kw) OR (('patients' NEAR/7 'activation'):ti,ab,kw) OR (('patients' NEAR/7 'activations'):ti,ab,kw) OR (('activating' NEAR/7 'patient'):ti,ab,kw) OR (('stakeholder' NEAR/7 'panel'):ti,ab,kw) OR (('stakeholder' NEAR/7 'panels'):ti,ab,kw) OR (('stakeholders' NEAR/7 'panels'):ti,ab,kw) OR (('stakeholders' NEAR/7 'panel'):ti,ab,kw) OR (('stakeholder' NEAR/7 'forum'):ti,ab,kw) OR (('stakeholder' NEAR/7 'forums'):ti,ab,kw) OR (('stakeholders' NEAR/7 'forums'):ti,ab,kw) OR (('stakeholders' NEAR/7 'forum'):ti,ab,kw) OR (('stakeholder' NEAR/7 'consultation'):ti,ab,kw) OR (('stakeholder' NEAR/7 'consultations'):ti,ab,kw) OR (('stakeholders' NEAR/7 'consultations'):ti,ab,kw) OR (('stakeholders' NEAR/7 'consultation'):ti,ab,kw) OR (('consulting' NEAR/7 'stakeholder'):ti,ab,kw) OR (('consulting' NEAR/7 'stakeholders'):ti,ab,kw) OR (('stakeholder' NEAR/7 'participation'):ti,ab,kw) OR (('stakeholder' NEAR/7 'participations'):ti,ab,kw) OR (('stakeholders' NEAR/7 'participation'):ti,ab,kw) OR (('participating' NEAR/7 'stakeholder'):ti,ab,kw) OR (('participating' NEAR/7 'stakeholders'):ti,ab,kw) OR (('stakeholder' NEAR/7 'involvement'):ti,ab,kw) OR (('stakeholders' NEAR/7 'involvements'):ti,ab,kw) OR (('stakeholders' NEAR/7 'involvement'):ti,ab,kw) OR (('stakeholder' NEAR/7 'involvements'):ti,ab,kw) OR (('involving' NEAR/7 'stakeholder'):ti,ab,kw) OR (('involving' NEAR/7 'stakeholders'):ti,ab,kw) OR (('stakeholder' NEAR/7 'engagement'):ti,ab,kw) OR (('stakeholders' NEAR/7 'engagement'):ti,ab,kw) OR (('stakeholder' NEAR/7 'engagements'):ti,ab,kw) OR (('stakeholders' NEAR/7 'engagements'):ti,ab,kw) OR (('engaging' NEAR/7 'stakeholder'):ti,ab,kw) OR (('engaging' NEAR/7 'stakeholders'):ti,ab,kw) OR (('stakeholder' NEAR/7 'network'):ti,ab,kw) OR (('stakeholder' NEAR/7 'networks'):ti,ab,kw) OR (('stakeholders' NEAR/7 'network'):ti,ab,kw) OR (('stakeholders' NEAR/7 'networks'):ti,ab,kw) OR (('stakeholder' NEAR/7 'networking'):ti,ab,kw) OR (('stakeholders' NEAR/7 'networking'):ti,ab,kw) OR (('stakeholder' NEAR/7 'activation'):ti,ab,kw) OR (('stakeholder' NEAR/7 'activations'):ti,ab,kw) OR (('stakeholders' NEAR/7 'activation'):ti,ab,kw) OR (('stakeholders' NEAR/7 'activations'):ti,ab,kw) OR (('activating' NEAR/7 'stakeholder'):ti,ab,kw) OR (('society' NEAR/7 'panel'):ti,ab,kw) OR (('society' NEAR/7 'panels'):ti,ab,kw) OR (('society' NEAR/7 'forum'):ti,ab,kw) OR (('society' NEAR/7 'forums'):ti,ab,kw) OR (('society' NEAR/7 'consultation'):ti,ab,kw) OR (('society' NEAR/7 'consultations'):ti,ab,kw) OR (('society' NEAR/7 'consulting'):ti,ab,kw) OR (('society' NEAR/7 'participation'):ti,ab,kw) OR (('society' NEAR/7 'participations'):ti,ab,kw) OR (('society' NEAR/7

'participating'):ti,ab,kw) OR (('society' NEAR/7 'involvement'):ti,ab,kw) OR (('society' NEAR/7 'involvements'):ti,ab,kw) OR (('society' NEAR/7 'involving'):ti,ab,kw) OR (('society' NEAR/7 'engagement'):ti,ab,kw) OR (('society' NEAR/7 'engagements'):ti,ab,kw) OR (('society' NEAR/7 'engaging'):ti,ab,kw) OR (('society' NEAR/7 'network'):ti,ab,kw) OR (('society' NEAR/7 'networks'):ti,ab,kw) OR (('society' NEAR/7 'activation'):ti,ab,kw) OR (('society' NEAR/7 'activations'):ti,ab,kw) OR (('society' NEAR/7 'activating'):ti,ab,kw) OR (('needs' NEAR/7 'assessment'):ti,ab,kw) OR (('need' NEAR/7 'assessment'):ti,ab,kw) OR (('needs' NEAR/7 'assessments'):ti,ab,kw) OR (('need' NEAR/7 'assessments'):ti,ab,kw) OR (('assessing' NEAR/7 'need'):ti,ab,kw) OR (('assessing' NEAR/7 'needs'):ti,ab,kw) OR (('need' NEAR/7 'determination'):ti,ab,kw) OR (('determining' NEAR/7 'need'):ti,ab,kw) OR (('needs' NEAR/7 'determination'):ti,ab,kw) OR (('determining' NEAR/7 'needs'):ti,ab,kw) OR (('need' NEAR/7 'determinations'):ti,ab,kw) OR (('needs' NEAR/7 'determinations'):ti,ab,kw) OR 'patient$ outcome$':ti,ab,kw OR (('epidemiologic' NEAR/7 'research'):ti,ab,kw) OR (('epidemiological' NEAR/7 'research'):ti,ab,kw) OR (('epidemiologic' NEAR/7 'method'):ti,ab,kw) OR (('epidemiological' NEAR/7 'methods'):ti,ab,kw) OR (('epidemiologic' NEAR/7 'study'):ti,ab,kw) OR (('epidemiological' NEAR/7 'study'):ti,ab,kw) OR (('epidemiologic' NEAR/7 'studies'):ti,ab,kw) OR (('epidemiological' NEAR/7 'studies'):ti,ab,kw) OR (('health services' NEAR/7 'research'):ti,ab,kw) OR (('health service' NEAR/7 'research'):ti,ab,kw) OR (('health services' NEAR/7 'evaluation'):ti,ab,kw) OR (('health services' NEAR/7 'evaluations'):ti,ab,kw) OR (('health service' NEAR/7 'evaluation'):ti,ab,kw) OR (('health service' NEAR/7 'evaluations'):ti,ab,kw) OR (('health care' NEAR/7 'research'):ti,ab,kw) OR (('healthcare' NEAR/7 'research'):ti,ab,kw) OR (('observational' NEAR/7 'research'):ti,ab,kw) OR (('observational' NEAR/7 'studies'):ti,ab,kw) OR (('observational' NEAR/7 'study'):ti,ab,kw) OR (('cohort' NEAR/7 'research'):ti,ab,kw) OR (('cohort' NEAR/7 'study'):ti,ab,kw) OR (('cohort' NEAR/7 'studies'):ti,ab,kw) OR (('case-control' NEAR/7 'study'):ti,ab,kw) OR (('case-control' NEAR/7 'studies'):ti,ab,kw) OR (('healthcare' NEAR/7 'evaluation'):ti,ab,kw) OR (('health care' NEAR/7 'evaluation'):ti,ab,kw) OR (('health-care' NEAR/7 'evaluation'):ti,ab,kw))

AND

('unmet medical need'/exp OR (('unmet' NEAR/7 'need'):ti,ab,kw) OR (('unmet' NEAR/7 'needs'):ti,ab,kw) OR (('unmet' NEAR/7 'demand'):ti,ab,kw) OR (('unmet' NEAR/7 'demands'):ti,ab,kw) OR (('societal' NEAR/7 'need'):ti,ab,kw) OR (('societal' NEAR/7 'needs'):ti,ab,kw) OR (('societal' NEAR/7 'demand'):ti,ab,kw) OR (('societal' NEAR/7 'demands'):ti,ab,kw) OR (('society' NEAR/7 'need'):ti,ab,kw) OR (('society' NEAR/7 'needs'):ti,ab,kw) OR (('society' NEAR/7 'demand'):ti,ab,kw) OR (('society' NEAR/7 'demands'):ti,ab,kw) OR (('community' NEAR/7 'need'):ti,ab,kw) OR (('community' NEAR/7 'needs'):ti,ab,kw) OR (('community' NEAR/7 'demand'):ti,ab,kw) OR (('community' NEAR/7

'demands'):ti,ab,kw) OR (('communities' NEAR/7 'need'):ti,ab,kw) OR (('communities' NEAR/7 'needs'):ti,ab,kw) OR (('communities' NEAR/7 'demand'):ti,ab,kw) OR (('communities' NEAR/7 'demands'):ti,ab,kw) OR (('health' NEAR/7 'need'):ti,ab,kw) OR (('health' NEAR/7 'needs'):ti,ab,kw) OR (('health' NEAR/7 'demand'):ti,ab,kw) OR (('health' NEAR/7 'demands'):ti,ab,kw) OR (('healthcare' NEAR/7 'need'):ti,ab,kw) OR (('healthcare' NEAR/7 'needs'):ti,ab,kw) OR (('healthcare' NEAR/7 'demand'):ti,ab,kw) OR (('healthcare' NEAR/7 'demands'):ti,ab,kw) OR (('health care' NEAR/7 'need'):ti,ab,kw) OR (('health care' NEAR/7 'needs'):ti,ab,kw) OR (('health care' NEAR/7 'demand'):ti,ab,kw) OR (('health care' NEAR/7 'demands'):ti,ab,kw) OR (('care' NEAR/7 'need'):ti,ab,kw) OR (('care' NEAR/7 'needs'):ti,ab,kw) OR (('care' NEAR/7 'demand'):ti,ab,kw) OR (('care' NEAR/7 'demands'):ti,ab,kw) OR (('medical' NEAR/7 'need'):ti,ab,kw) OR (('medical' NEAR/7 'needs'):ti,ab,kw) OR (('medical' NEAR/7 'demand'):ti,ab,kw) OR (('medical' NEAR/7 'demands'):ti,ab,kw) OR (('therapeutic' NEAR/7 'need'):ti,ab,kw) OR (('therapeutic' NEAR/7 'needs'):ti,ab,kw) OR (('therapeutic' NEAR/7 'demand'):ti,ab,kw) OR (('therapeutic' NEAR/7 'demands'):ti,ab,kw) OR (('therapeutical' NEAR/7 'need'):ti,ab,kw) OR (('therapeutical' NEAR/7 'needs'):ti,ab,kw) OR (('therapeutical' NEAR/7 'demand'):ti,ab,kw) OR (('therapeutical' NEAR/7 'demands'):ti,ab,kw) OR (('pharmaceutic' NEAR/7 'need'):ti,ab,kw) OR (('pharmaceutic' NEAR/7 'needs'):ti,ab,kw) OR (('pharmaceutic' NEAR/7 'demand'):ti,ab,kw) OR (('pharmaceutic' NEAR/7 'demands'):ti,ab,kw) OR (('pharmaceutical' NEAR/7 'need'):ti,ab,kw) OR (('pharmaceutical' NEAR/7 'needs'):ti,ab,kw) OR (('pharmaceutical' NEAR/7 'demand'):ti,ab,kw) OR (('pharmaceutical' NEAR/7 'demands'):ti,ab,kw) OR (('patient' NEAR/7 'need'):ti,ab,kw) OR (('patient' NEAR/7 'needs'):ti,ab,kw) OR (('patient' NEAR/7 'demand'):ti,ab,kw) OR (('patient' NEAR/7 'demands'):ti,ab,kw) OR (('patients' NEAR/7 'need'):ti,ab,kw) OR (('patients' NEAR/7 'needs'):ti,ab,kw) OR (('patients' NEAR/7 'demand'):ti,ab,kw) OR (('patients' NEAR/7 'demands'):ti,ab,kw) OR (('population' NEAR/7 'need'):ti,ab,kw) OR (('population' NEAR/7 'needs'):ti,ab,kw) OR (('population' NEAR/7 'demand'):ti,ab,kw) OR (('population' NEAR/7 'demands'):ti,ab,kw) OR (('populations' NEAR/7 'need'):ti,ab,kw) OR (('populations' NEAR/7 'needs'):ti,ab,kw) OR (('populations' NEAR/7 'demand'):ti,ab,kw) OR (('populations' NEAR/7 'demands'):ti,ab,kw) OR (('therapy' NEAR/7 'need'):ti,ab,kw) OR (('therapy' NEAR/7 'needs'):ti,ab,kw) OR (('therapy' NEAR/7 'demand'):ti,ab,kw) OR (('therapy' NEAR/7 'demands'):ti,ab,kw))

AND

(‘pediatrics’/exp OR ‘p$ediatrics’:ti,ab,kw OR ‘neonat*’:ti,ab,kw OR ‘child’/exp OR ‘child*’:ti,ab,kw ‘hospitalized child’/exp OR ‘juvenile’:ti,ab,kw OR ‘infan*’:ti,ab,kw OR ‘toddler*’:ti,ab,kw OR ‘baby’:ti,ab,kw OR ‘babies’:ti,ab,kw OR ‘newborn*’:ti,ab,kw OR ‘adolescent’/de OR ‘hospitalized adolescent’/exp OR ‘adolescen*’:ti,ab,kw OR ‘perinat*’:ti,ab,kw OR ‘minor person’/exp OR

‘minors’:ti,ab,kw OR ‘teen*’:ti,ab,kw OR ‘youth*’:ti,ab,kw OR ((‘1’ OR ‘2’ OR ‘3’ OR ‘4’ OR ‘5’ OR ‘6’ OR ‘7’ OR ‘8’ OR ‘9’ OR ‘10’ OR ‘11’ OR ‘12’ OR ‘13’ OR ‘14’ OR ‘15’ OR ‘16’ OR ‘one’ OR ‘two’ OR ‘three’ OR ‘four’ OR ‘five’ OR ‘six’ OR ‘seven’ OR ‘eight’ OR ‘nine’ OR ‘ten’ OR ‘eleven’ OR ‘twelve’ OR ‘thirteen’ OR ‘fourteen’ OR ‘fifteen’ OR ‘sixteen’ OR ‘17’ OR ‘18’ OR ‘19’ OR ‘20’ OR ‘21’ OR ‘22’ OR ‘23’ OR ‘24’ OR ‘25’ OR ‘26’ OR ‘27’ OR ‘28’ OR ‘29’ OR ‘30’ OR ‘31’ OR ‘32’ OR ‘33’ OR ‘34’) NEXT/2 (‘week*’) AND (‘old’ OR ‘age’ OR ‘aged’ OR ‘ages’)):ti,ab,kw OR ((‘1’ OR ‘2’ OR ‘3’ OR ‘4’ OR ‘5’ OR ‘6’ OR ‘7’ OR ‘8’ OR ‘9’ OR ‘10’ OR ‘11’ OR ‘12’ OR ‘13’ OR ‘14’ OR ‘15’ OR ‘16’ OR ‘one’ OR ‘two’ OR ‘three’ OR ‘four’ OR ‘five’ OR ‘six’ OR ‘seven’ OR ‘eight’ OR ‘nine’ OR ‘ten’ OR ‘eleven’ OR ‘twelve’ OR ‘thirteen’ OR ‘fourteen’ OR ‘fifteen’ OR ‘sixteen’ OR ‘17’ OR ‘18’ OR ‘19’ OR ‘20’ OR ‘21’ OR ‘22’ OR ‘23’ OR ‘24’) NEXT/2 (‘month*’) AND (‘old’ OR ‘age’ OR ‘aged’ OR ‘ages’)):ti,ab,kw OR ((‘1’ OR ‘2’ OR ‘3’ OR ‘4’ OR ‘5’ OR ‘6’ OR ‘7’ OR ‘8’ OR ‘9’ OR ‘10’ OR ‘11’ OR ‘12’ OR ‘13’ OR ‘14’ OR ‘15’ OR ‘16’ OR ‘one’ OR ‘two’ OR ‘three’ OR ‘four’ OR ‘five’ OR ‘six’ OR ‘seven’ OR ‘eight’ OR ‘nine’ OR ‘ten’ OR ‘eleven’ OR ‘twelve’ OR ‘thirteen’ OR ‘fourteen’ OR ‘fifteen’ OR ‘sixteen’ OR ‘17’ OR ‘18’) NEXT/2 (‘year*’) AND (‘old’ OR ‘age’ OR ‘aged’ OR ‘ages’)):ti,ab,kw OR ‘day* old’:ti,ab,kw OR ‘month* old’:ti,ab,kw OR ‘kid’:ti,ab,kw OR ‘kids’:ti,ab,kw OR ‘puberty’/exp OR ‘puber*’:ti,ab,kw OR ‘prepuber*’:ti,ab,kw OR ‘menarche’:ti,ab,kw OR ‘school age*’:ti,ab,kw OR ‘schoolchild*’:ti,ab,kw OR ‘preschool*’:ti,ab,kw OR ‘preteen*’:ti,ab,kw OR ‘youngster*’:ti,ab,kw OR ‘first grade*’:ti,ab,kw OR ‘second grade*’:ti,ab,kw OR ‘third grade*’:ti,ab,kw OR ‘fourth grade*’:ti,ab,kw OR ‘fifth grade*’:ti,ab,kw OR ‘sixth grade*’:ti,ab,kw OR ‘seventh grade*’:ti,ab,kw OR ‘eight grade*’:ti,ab,kw OR ‘ninth grade*’:ti,ab,kw OR ‘tenth grade*’:ti,ab,kw OR ‘eleventh grade*’:ti,ab,kw OR ‘twelfth grade*’:ti,ab,kw OR ‘grade 1’:ti,ab,kw OR ‘grade 2’:ti,ab,kw OR ‘grade 3’:ti,ab,kw OR ‘grade 4’:ti,ab,kw OR ‘grade 5’:ti,ab,kw OR ‘grade 6’:ti,ab,kw OR ‘grade 7’:ti,ab,kw OR ‘grade 8’:ti,ab,kw OR ‘grade 9’:ti,ab,kw OR ‘grade 10’:ti,ab,kw OR ‘grade 11’:ti,ab,kw OR ‘grade 12’:ti,ab,kw OR ‘grade one’:ti,ab,kw OR ‘grade two’:ti,ab,kw OR ‘grade three’:ti,ab,kw OR ‘grade four’:ti,ab,kw OR ‘grade five’:ti,ab,kw OR ‘grade six’:ti,ab,kw OR ‘grade seven’:ti,ab,kw OR ‘grade eight’:ti,ab,kw OR ‘grade nine’:ti,ab,kw OR ‘grade ten’:ti,ab,kw OR ‘grade eleven’:ti,ab,kw OR ‘grade twelve’:ti,ab,kw OR ‘nursery’:ti,ab,kw OR ‘low birth weight’/exp OR ‘low birth weight’:ti,ab,kw OR ‘LBW’:ti,ab,kw OR ‘VLBW’:ti,ab,kw OR ‘ELBW’:ti,ab,kw OR ‘VPT’:ti,ab,kw OR ‘birth’:ti,ab,kw OR ‘birth’/de OR ‘childbirth’:ti,ab,kw OR ‘born’:ti,ab,kw OR ‘immatur*’:ti,ab,kw OR ‘NICU’:ti,ab,kw OR ‘postnat*’:ti,ab,kw OR ‘post-nat*’:ti,ab,kw OR ‘prematur*’:ti,ab,kw OR ‘pre-nat*’:ti,ab,kw OR ‘preterm’:ti,ab,kw OR ‘pre-term’:ti,ab,kw OR ‘congenital’:ti,ab,kw)

AND

(‘leukemia’/exp OR ‘Leuk$emia*’:ti,ab,kw OR ‘AML’:ti,ab,kw OR ‘MPAL*’:ti,ab,kw OR ‘APL’:ti,ab,kw OR ‘CML’:ti,ab,kw OR ‘JMML’:ti,ab,kw OR ‘Leucocyth$emia*’:ti,ab,kw OR ‘Lymphoma’/exp OR ‘lymphoma*’:ti,ab,kw OR ‘non-Hodgkin’:ti,ab,kw OR ‘Hodgkin’:ti,ab,kw OR ‘PMBL’:ti,ab,kw OR ‘HL’:ti,ab,kw OR ‘NSCHL’:ti,ab,kw OR ‘NLPHL’:ti,ab,kw OR ‘MCCHL’:ti,ab,kw OR ‘NHL’:ti,ab,kw OR ‘LBL’:ti,ab,kw OR ‘Burkitt’:ti,ab,kw OR ‘DLBCL’:ti,ab,kw OR ‘ALCL’:ti,ab,kw OR ((‘blood’:ti,ab,kw OR ‘h$ematologic’:ti,ab,kw) AND (‘cancer*’:ti,ab,kw OR ‘tumo$r*’:ti,ab,kw OR ‘neoplasm*’:ti,ab,kw OR ‘carcinoma*’:ti,ab,kw)))

Supplementary Information II: data extraction framework

General information

- Article title
- Author names
- Author affiliation
- Publication year
- Geographical region
- Broad method category
- Specific method category
- Measure/tool (methodological instrument)
- Study participant group
- Number of participants
- Participant details
- Age of participants

Patient health needs

- Impact on general health-related QOL
- Burden of disease
- Dental care
- Impact on physical activity
- Impact on development
- Impact on mental health
- Impact on autonomy

Patient healthcare needs

- Burden of treatment
- Experienced quality of care

Patient social needs

- Impact on social life
- Impact on future perspectives
- Impact on education
- Financial consequences

Burden on informal caregiver (parent)

- Impact on physical health
- Impact on psychological health
- Impact on social life
- Information needs
- Experienced quality of care
- Financial consequences

Burden on sibling

- Impact on social life of sibling
- Information needs of siblings

Supplementary materials III: summary of included studies

Article	Geographical region	Method	Participant	Criteria	Tool
**Wills, 1999**	Europe	Interview	Parent	• Impact on mental health family	
**Collards, 2001**	Europe	Interview	Parent and patient	• Forgone care<br>• Impact on mental health of family	
**Theunissen, 2007**	Europe	Questionnaire	Parent	• Burden of disease symptoms<br>• Impact on autonomy and development<br>• Impact on mental health of patient<br>• Impact on social life of patient<br>• Impact on education<br>• impact on mental health of informal caregiver<br>• financial consequences	
**Cubukçu, 2008**	Asia	Interview		• Forgone care	
**Hutton, 2009**	Europe	Clinical investigation	Patient	• Forgone care	

**Reinfjell, 2009**	Europe	Questionnaire	Parent and patient	• Impact on mental health of the patient	• CBCL<br>• YSR<br>• SDQ
**Wills, 2009**	North-America	Interview	Parent	• Information needs of patient<br>• Impact on mental health of family	
**Keegan, 2012**	North-America	Questionnaire	Patient	• Impact on mental health of patient<br>• Information needs of patient	
**Zebrack, 2012**	North-America	Questionnaire	Patient	• Impact on autonomy and development<br>• Impact on mental health of patient	
**Cepuch, 2013**	Europe	Questionnaire	Parent	• Impact on mental health of family	• PSS-10
**Yildirim, 2013**	Asia	Interview	Parent	• Burden of treatment side-effects<br>• Impact on social life of patient	
**Götte, 2014**	Europe	Interview	Patient	• Impact on physical activity	

				• Impact on autonomy and development<br>• Experiences burden of side effects<br>• Impact on social life of patient	
**Muskat, 2017**	North-America	Interview	Parent	• Impact on mental health of family	
**Tenniglo, 2017**	Europe	Focusgroup	Parent and patient	• Involvement of patients<br>• Impact on education<br>• Experiences with healthcare organisation and HCP by family	
**Hocking, 2018**	North-America	Questionnaire	Parent	• Impact on education	
**Beentjes, 2018**	Europe	Review	Patient	• Impact on autonomy and development<br>• Impact on mental health of patient<br>• Information needs of patient<br>• Involvement of patients<br>• Impact on social life of patients	

				• Impact on mental health of family	
**Ding, 2019**	North-America	Interview	Parent and patient	• Involvement of patients<br>• Financial consequences	
**Lee, 2020**	Asia	Interview	Parent	• Impact on education<br>• Impact on mental health of informal caregiver<br>• Financial consequences	
**Andrés-Jensen, 2020**	Europe	Interview	Patient	• Impact on social life of patient<br>• Impact on education	
**Lövgren, 2020**	Europe	Questionnaire	Other	• Information needs of informal caregiver<br>• Information needs of siblings	FACES-IV
**Liu Q, 2021**	Asia	Interview	Parent	• Impact on mental health of family<br>• Financial consequences	
**Liu Y, 2021**	Asia	Questionnaire	Parent	• Impact on mental health of informal caregiver<br>• Financial consequences	
**Saritshasombat, 2021**	Asia	Interview	Patient	• Information needs of patient	

				• Impact on social life of patient<br>• Impact on education of patient	
**Sopfe, 2021**	North-America	Interview	Patient	• Information needs of patient	
**Al-Dhawyani, 2022**	Asia	Questionnaire	Parent	• Impact on mental health of family<br>• Information needs family	
**Chong, 2022**	Asia	Interview	Parent	• Impact on mental health of informal caregiver	
**Ding, 2022**	North-America	Interview	Parent and patient	• Involvement of patients<br>• Financial consequence	
**Huang, 2022**	Asia	Interview	Parent	• Impact on mental health of informal caregiver<br>• Information needs of informal caregiver<br>• Impact on social life of the siblings<br>• Financial consequences	
**Kastrinos, 2022**	North-America	Interview	Parent	• Information needs of informal caregiver	

				• Impact on social life of the siblings	
**Chodidjah, 2022**	Asia	Interview	Other	• Impact on mental health<br>• Treatment adherence<br>• Impact on mental health of informal caregiver<br>• Impact on social life of informal caregiver	
**Barton, 2023**	North-America	Interview	Patient	• Impact on mental health of patient	
**Paterson, 2023**	Intercontinental	Review	Patient	• Impact on mental health of patient<br>• Experienced burden of side effects<br>• Experiences with healthcare organisations and HCP<br>• Information needs of patient<br>• Impact on social life of patient<br>• Impact on education	

**Stenmarker, 2023**	Europe	Mixed: interview + literature	Patient	• Experiences with healthcare organisations and HCP	
**Leibring, 2024**	Europe	Interview	Patient	• Impact on autonomy and development<br>• Impact on mental health of patient<br>• Impact on social life of patient<br>• Impact on future perspectives<br>• Impact on education	
